# Identification and manipulation of the pleuromutilin gene cluster from *Clitopilus passeckerianus* for increased rapid antibiotic production

**DOI:** 10.1038/srep25202

**Published:** 2016-05-04

**Authors:** Andy M. Bailey, Fabrizio Alberti, Sreedhar Kilaru, Catherine M. Collins, Kate de Mattos-Shipley, Amanda J. Hartley, Patrick Hayes, Alison Griffin, Colin M. Lazarus, Russell J. Cox, Christine L. Willis, Karen O’Dwyer, David W. Spence, Gary D. Foster

**Affiliations:** 1 School of Biological Sciences, Life Sciences Building, University of Bristol, 24 Tyndall Avenue, Bristol, BS8 1TQ, UK; 2 GSK, Southdownview Way, Worthing, West Sussex, BN14 8QH, UK; 3 School of Chemistry, University of Bristol, Cantock’s Close Bristol, BS8 1TS, UK; 4 GSK, 1250 S. Collegeville Road, Collegeville, Pennsylvania, 19426-0989, United States

## Abstract

Semi-synthetic derivatives of the tricyclic diterpene antibiotic pleuromutilin from the basidiomycete *Clitopilus passeckerianus* are important in combatting bacterial infections in human and veterinary medicine. These compounds belong to the only new class of antibiotics for human applications, with novel mode of action and lack of cross-resistance, representing a class with great potential. Basidiomycete fungi, being dikaryotic, are not generally amenable to strain improvement. We report identification of the seven-gene pleuromutilin gene cluster and verify that using various targeted approaches aimed at increasing antibiotic production in *C. passeckerianus*, no improvement in yield was achieved. The seven-gene pleuromutilin cluster was reconstructed within *Aspergillus oryzae* giving production of pleuromutilin in an ascomycete, with a significant increase (2106%) in production. This is the first gene cluster from a basidiomycete to be successfully expressed in an ascomycete, and paves the way for the exploitation of a metabolically rich but traditionally overlooked group of fungi.

Antimicrobial resistance is now universally considered to be one of the greatest threats to human health. Continually emerging resistance has been confounded by a lack of investment into antibiotic research and development over recent decades. The World Health Organization’s (WHO) 2014 report on global surveillance of antimicrobial resistance has recently warned that without urgent, coordinated action, the world is heading towards a post-antibiotic era, in which common infections and minor injuries, which have been treatable for decades, may again be fatal.

A highly promising class of antibiotics for human therapeutics are the semi-synthetic pleuromutilin antibiotics. Pleuromutilin is a tricyclic diterpene produced by the basidiomycete *Clitopilus passeckerianus* and related basidiomycete fungi^1^, which was initially isolated and characterized by Kavanagh and co-workers in 1951^2^. The pleuromutilin derivatives tiamulin and valnemulin have been used in veterinary medicine for many years^3^, but an increasing need for new antibiotics has led to a surge in the development of pleuromutilin derivatives specifically for human use.

In 2007, retapamulin became the first semi-synthetic pleuromutilin antibiotic to be approved by the FDA and EMA and is recommended for the treatment of impetigo and infected small lacerations, abrasions and sutured wounds^4^. This represents the first antibiotic of a new class to be approved for use against topical infections in more than 30 years.

Nabriva Therapeutics AG, a biotechnology company focused on developing pleuromutilin antibiotics, has been developing novel pleuromutilin derivatives with potent and broad-spectrum activities and the desired pharmacokinetic characteristics for oral and intravenous (IV) administration. One derivative in particular, BC-3781 (lefamulin), is particularly promising and has recently been granted Qualified Infectious Disease Product (QIDP) as well as Fast Track status designation, for the treatment of community-acquired bacterial pneumonia (CABP) and acute bacterial skin and skin structure infections (ABSSSI). Lefamulin is soon to enter phase III clinical trials for the treatment of CABP, and also holds great potential as a treatment for hospital-acquired and ventilator-associated bacterial pneumonia (HABP/VABP), as well as ABSSSI, with potential in several other indications (such as sexually transmitted infections, including multidrug-resistant MDR gonorrhea and osteomyelitis).

There has also been increasing interest in pleuromutilin antibiotics as potential agents to treat multi-drug resistant (MDR-) and extensively drug resistant (XDR-) tuberculosis strains, which are increasingly difficult to treat^5^,^6^. Although the biochemical properties and mode of action of pleuromutilins are well understood^7^,^8^,^9^,^10^, and a partial chemical elucidation of the potential pathway has previously been proposed^11^, the complete biosynthesis of this increasingly important natural product has never been elucidated. As with most natural products, and particularly fungal natural products, the yields in the native host are very low, and the process of fermentation and isolation of the desired compounds can be logistically challenging. This is particularly true for basidiomycete fungi such as *Clitopilus passeckerianus*, where traditional strain improvement techniques such as random mutagenesis are rarely successful due to their dikaryotic nature. For these reasons, understanding the biosynthetic pathway of a natural product is often key to developing a strain which can be exploited successfully.

In this work we set out to identify the pleuromutilin gene cluster and identify a strain improvement approach. Initial attempts to improve pleuromutilin yields in *C. passeckerianus* were followed by the reconstruction of the biosynthetic pathway in the heterologous host *Aspergillus oryzae*. This represents the first cross-phylum heterologous expression of a basidiomycete gene cluster in an ascomycete host, so will not only increase the potential of the pleuromutilin antibiotics but may also help to inform the future discovery and exploitation of secondary metabolites from the rich but underexploited basidiomycete fungi.

## Results

### Identification of the candidate pleuromutilin gene cluster

In fungi, it is usual for the gene clusters encoding enzymes for diterpene biosynthesis to include a cluster-specific geranylgeranyl pyrophosphate synthetase (GGS). On this assumption, we used PCR with degenerate primers ggs27 and ggs29 to isolate GGS-related products from the genome of *C. passeckerianus*. Amplification products were cloned and sequenced. After taking into account allelic differences due to the dikaryotic nature of the fungus, four different GGS-related sequences were obtained and these were screened to determine which followed the predicted expression patterns for pleuromutilin production by RT-PCR and Northern analysis. RT-PCR excluded *ggs-1*, whilst Northern analysis showed that only *ggs-2* was highly expressed under pleuromutilin-producing conditions (Supplementary Figure 1). Southern blot analysis determined that *ggs-2* is only present once in the genome (Supplementary Figure 2A).

**Figure 1:**
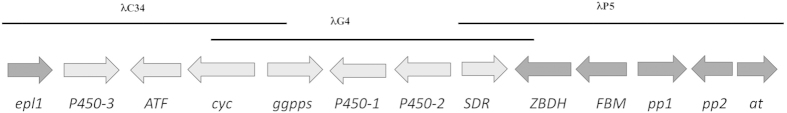
The pleuromutilin gene cluster of *C. passeckerianus*. Genes involved in the biosynthesis of pleuromutilin are light grey. Those in dark grey are considered to lie outside the gene cluster. The horizontal lines above show the regions of the cluster contained within the three lambda phage clones λC34, λG4 and λP5.

Probing a *C. passeckerianus* genomic library with this partial *ggs-2* PCR product identified lambda clone λG4, and sequencing the full 8.4 kb insert revealed a full-length *ggs* gene of 1291 bp (*Pl-ggs*), containing 4 putative introns and encoding a 350 amino-acid protein. Upstream of *Pl-ggs* was a partial diterpene cyclase (*cyc*) gene, named *Pl-cyc*; downstream was a full length cytochrome P450 gene (*Pl-p450-1*) and a second partial cytochrome P450 gene (*Pl-p450-2*), showing that *Pl-ggs* was located within a likely diterpene gene cluster. Rescreening the library to isolate contiguous sequences identified λC34, in which an overlapping region shared 100% homology with λG4, and λP5, whose overlap with λG4 contained several SNPs and indels. This indicated that λG4 and λC34 originate from the same allelic version of the pleuromutilin gene cluster, while λP5 is derived from the other allele. In total a genomic region of 34.6 kb was sequenced and 13 likely genes were identified (Fig. 1 and Table 1).

Whilst *Pl-ggs* and *Pl-cyc* could be inferred as the core genes of the diterpene synthesis, from bioinformatic analysis alone, it was not possible to confidently predict the boundaries of the gene cluster. We therefore analysed gene expression by northern blotting to identify which genes were coordinately expressed and correlated with conditions known to induce pleuromutilin biosynthesis.

Genes for GGS, cyclase, acetyl transferase (ATF), short-chain dehydrogenase/reductase (SDR) and three P450 s were shown to be upregulated during pleuromutilin production, whereas flavin binding monooxygenase (*fbm*), zinc binding dehydrogenase (*zbdh*), and aminotransferase genes were not, suggesting that they lie outside the pleuromutilin gene cluster (Supplementary Figure 3).

**Figure 2:**
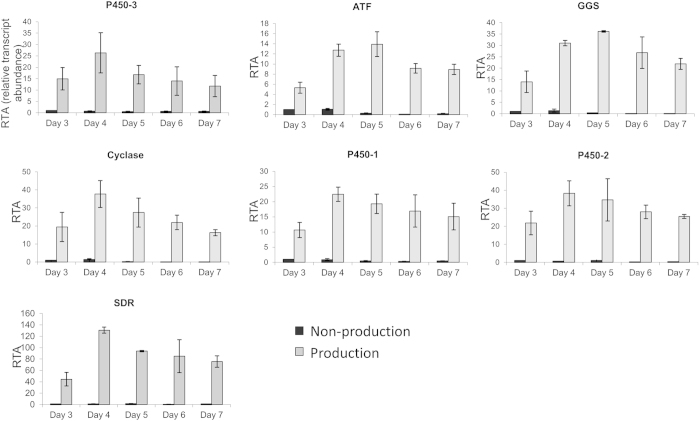
Real-time PCR data for the seven pleuromutilin biosynthesis genes from *C. passeckerianus* when grown in production (CGC) and non-production (MM) media. *β*-tubulin was used as a reference gene and non-production media on day 3 was used as the calibrator sample (all other expression is shown relative to this data point).

A more thorough expression analysis, combining quantitative RT-PCR with an analysis of pleuromutilin production, demonstrated that the seven putative pleuromutilin biosynthetic genes are not only highly differentially expressed under production and non-production conditions, but also tightly co-regulated (Fig. 2). See Supplementary Figure 4 for the corresponding time-course of pleuromutilin yields.

The gene for farnesyl diphosphate synthase (*fds*) was also isolated and sequenced to assess any role in the pathway. This is another key enzyme in isoprenoid biosynthesis which catalyzes the formation of farnesyl diphosphate, a precursor for several classes of essential metabolites. Degenerate primers (fdsf2A and fdsr4) were used to amplify an *fds* gene fragment, which was used to pull out a lambda clone that was sequenced in its entirety (Supplementary Figure 5). Southern blot analysis demonstrated that this *fds* is only present once in the genome, at a different locus to the pleuromutilin cluster, suggesting that it is responsible for the biosynthesis of farnesyl diphosphate for both primary and secondary metabolism. Genes encoding β-tubulin and α- actin were also isolated from a genomic library as reported in Kilaru *et al.*^12^, allowing their promoter elements to be exploited for use in expression cassettes.

A gene-silencing approach was used to directly link the 7-gene cluster with pleuromutilin production, where the two core genes *Pl-ggs and Pl-cyc* were silenced. Plasmids based on pYES-hph-cbxgene (Supplementary Figure 6) were generated containing the full length coding regions of *Pl-ggs and Pl-cyc* in the antisense direction, under the control of the *Agaricus bisporus gpdII* promoter. Silencing using antisense constructs has previously been demonstrated to be an efficient approach to genetic manipulation in *C. passeckerianus*^12^. Initial screening of transformants via plate-based bioassay revealed reduced clearing zones for both genes (Supplementary Figures 7 to 9), providing direct evidence for their involvement in pleuromutilin production. Further analysis of selected transformants by HPLC demonstrated reduction in pleuromutilin titres. For example the yield of pleuromutilin was reduced by 87% to 103 μg/g in transformant p004-GGSantigene-16, compared to the wild-type *C. passeckerianus* yield of 765 μg/g.

### Attempted increase in pleuromutilin production in *C. passeckerianus*

Overexpression of the putative pleuromutilin biosynthetic genes was attempted in *C. passeckerianus*. Initially, the native promoters were used to drive expression. Eight expression vectors were constructed (Supplementary Figure 6B), containing the genes *Pl-p450-3*, *Pl-atf*, *Pl-cyc*, *Pl-ggs*, *Pl-p450-1*, *Pl-p450-2*, *Pl-sdr*, and *Cp-fbm*, and transformed into *C. passeckerianus* (Table 2). At least fifty transformants for each gene were screened *via* plate-based bioassay with *Bacillus subtilis* but no set of transformants showed any significant increase in clearing zone compared to untransformed *C. passeckerianus* (Supplementary Figures 10 to 17). Unexpectedly, however, every set of transformants contained some strains which exhibited a significant reduction of clearing zone, with some transformants expressing *Pl-ggs*, *Pl-cyc* or *Pl-p450-1* showing a complete disappearance of antibacterial activity. In particular, overexpression of *Pl-cyc* from its native promoter generated a range of transformants with greatly reduced or complete absence of clearing zones (Supplementary Figure 11). An HPLC analysis of pYES-hph-nativeCycgene transformants 2, 12, 23 and 43 showed a reduction in pleuromutilin titres of 38, 75, 98 and 89% respectively (436, 177, 11 and 78 μg/g) when compared to wild type *C. passeckerianus* (703 μg/g). This suggests that a form of sense suppression is occurred in these transformants.

As overexpression using native promoters did not enhance pleuromutilin production, the constitutive strong promoter *gpdII* from *A. bisporus* was instead used to drive expression of six genes in *C. passeckerianus*. Twelve constructs were made to include the individual genes *Cp-fds*, *Pl-ggs*, *Pl-cyc*, *Pl-p450-1, Pl-p450-2*, and *Pl-p450-3*, either with or without an additional intron (64 bp intron-exon region of *A. bisporus gpdII*) at the 5′ end of the gene (Supplementary Figure 6C). The presence of a 5′ intron has previously been shown to be essential for successful green fluorescent protein (*gfp*) and phleomycin resistance (*ble*) gene expression in *C. passeckerianus*^12^. At least twenty transformants were generated for each group using the 12 expression vectors (Table 3). Transformants overexpressing the *Cp-fds* or *Pl-ggs* were screened using HPLC for improved pleuromutilin titres. The majority of transformants demonstrated no significant increase in antibiotic titres, but one strain expressing the *Pl-ggs* without the additional intron showed a 50% increase in pleuromutilin yields when compared to wild-type *C. passeckerianus* (Table 2 and Supplementary Figure 18). Northern blot analysis showed increased levels of *Pl-ggs* transcripts when compared to the wild-type (Supplementary Figure 19), suggesting that the improved titre is due to increased *GGS* expression. The strains overexpressing *Pl-cyc*, *Pl-p450-1, Pl-p450-2*, or *Pl-p450-3* were screened *via* plate-based bioassay against *B. subtilis*, but no significant increases in clearing zones were observed (Table 3 and Supplementary Figures 20 to 26). A reduction in clearing zone diameter was again observed for some transformants from every group, and a complete loss of observable inhibition was seen for one transformant expressing the *Cyclase* gene without the additional intron (Supplementary Figure 20).

As a final approach to increase pleuromutilin titres in the native host, the entire putative gene cluster for pleuromutilin – comprising the nine genes *Pl-p450-3*, *Pl-atf*, *Pl-cyc*, *Pl-ggs*, *Pl-p450-1*, *Pl-p450-2*, *Pl-sdr*, *Cp-zbdh* and *Cp-fbm* – was cloned into a yeast shuttle vector (pYES2-hph-pleurocluster – Supplementary Figure 6D), and transformed into *C. passeckerianus*. One hundred and nineteen transformants were screened for increased antibiotic production by plate-based bioassay. Sixteen transformants showed a 20 to 40% increase in clearing zone diameter, but seven transformants showed complete disappearance of clearing zone (Supplementary Figures 27 and 28).

### Heterologous production of pleuromutilin in *A. oryzae*

**Figure 3:**
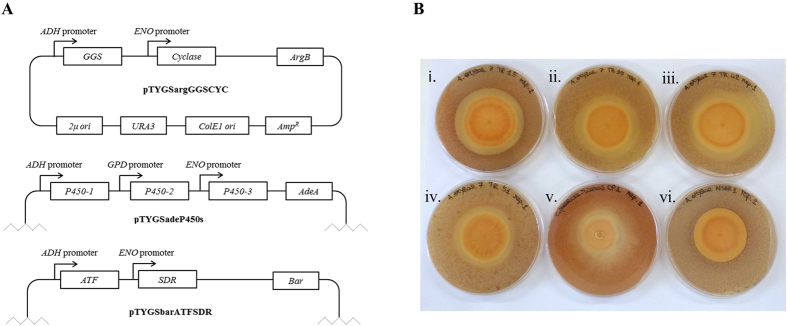
(**A**) Plasmid maps of vectors pTYGSargGGSCYC, pTYGSadeP450s, pTYGSbarATFSDR. The features for replication and selection in *E. coli* and *S. cerevisiae* are common to the three vectors, therefore shown only in pTYGSargGGSCYC. (**B**) Plate-based bioassay showing clearing zones produced by (i) *A. oryzae* NSAR1 7 TR15, (ii) *A. oryzae* NSAR1 7 TR35, (iii) *A. oryzae* NSAR1 7 TR42, (iv) *A. oryzae* NSAR1 7 TR51, (v) *C. passeckerianus* CP1, and (vi) untransformed *A. oryzae* NSAR1.

**Figure 4:**
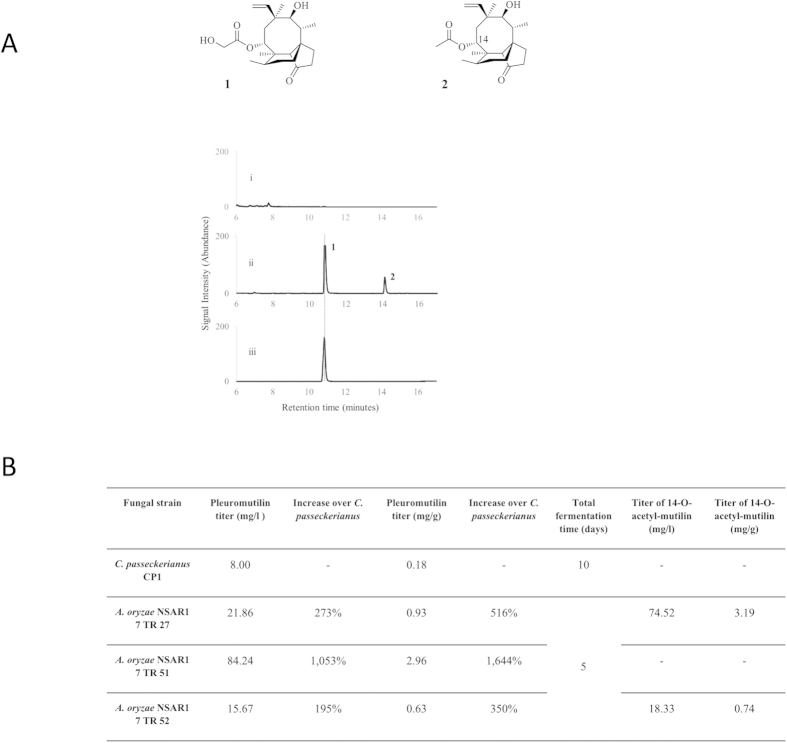
(**A)** *De novo* production of pleuromutilin **(1)** and 14-*O*-acetyl-mutilin **(2)** in *A. oryzae*. HPLC traces of untransformed *A. oryzae* NSAR1 (i), *A. oryzae* with *GGS*, *Cyclase*, *P450-1*, *P450-2*, *P450-3*, *ATF*, and *SDR* from *C. passeckerianus* (ii), and authentic pleuromutilin (iii). All traces were monitored through ELSD (Evaporative Light Scattering Detector). (**B)** Pleuromutilin and 14-O-acetyl-mutilin titres from *A. oryzae* transformant strains and *C. passeckerianus*. The titres are expressed either as mg of compound produced per litre of fungal culture (mg/l) or as mg of compound produced per gram of dry mass of mycelium (mg/g). *C. passeckerianus* required a total of 10 days between inoculation and harvesting, due to the need for a 5-day seed culture in PVS, followed by another 5-day production culture in CGC. *A. oryzae* transformants were only grown for 5 days in production culture CMP, which was inoculated directly from plates.

We heterologously expressed the seven genes of the putative gene cluster (*Pl-p450-3*, *Pl-atf*, *Pl-cyc*, *Pl-ggs*, *Pl-p450-1*, *Pl-p450-2*, *Pl-sdr*) in *A. oryzae* NSAR1, using our established *Aspergillus oryzae* multi-gene expression system^13^. Each of the genes were amplified from cDNA and placed under the control of *A. oryzae* promoters (Fig. 3A). Twelve independent transformants were screened for their ability to inhibit *B. subtilis* growth *via* plate-based bioassay. Eleven strains showed production and diffusion of an antibacterial compound in the medium, witnessed by appearance of clearing zone around the mycelia (Fig. 3B; Supplementary Figure 29). Only one strain (NSAR1 7 TR49) did not inhibit bacterial growth. RT-PCR demonstrated that three out of the seven transgenes were not being expressed in this strain (*Pl-p450-1*, *Pl-p450-2* and *Pl-p450-3*). Expression of all seven transgenes was confirmed for all of the other transformants (Supplementary Figure 30). To confirm pleuromutilin production, fermentation cultures were extracted with ethyl acetate, and the resulting crude extracts were analysed by HPLC-MS. As expected no production of new metabolites was detected from strain NSAR1 7 TR49, whereas two new compounds (**1** and **2**) were detected from all other strains (Fig. 4A), with **1** showing the same retention time and mass/charge ratio (*m/z*) as authentic pleuromutilin. **1** and **2** were purified from a 1 L culture *via* preparative-HPLC (yielding 12.4 mg and 7.5 mg respectively), and characterized by NMR spectroscopy and ESIHRMS. **1** was determined to be pleuromutilin, by ESIHRMS and by a comparison of the ^1^H-NMR and ^13^C-NMR data with that of authentic pleuromutilin and data in the literature^14^ (Supplementary Figures 31 to 34; Supplementary Table 2). ESIHRMS and a full-range of NMR analyses – including 2D-COSY, HMBC, and HSQC – allowed the identification of **2** as 14-O-acetyl-mutilin (Supplementary Figures 35 to 39; Supplementary Table 3), which is another known product of fermentation of *C. passeckerianus*^15^. It has not previously been proven to a precursor of pleuromutilin.

Pleuromutilin yields were quantified for the *A. oryzae* transformants showing the highest production of **1** from the analytical HPLC-MS, and a 195 to 1,053% increase over the native producer was observed, with a maximum production of 84.24 mg/l for NSAR1 7 TR51 (Fig. 4B). Taking into account that *C. passeckerianus* requires a preliminary seed culture prior to fermentation, the time between initial culture inoculation and harvesting of the antibiotic is also halved in *A. oryzae* compared to *C. passeckerianus* (Fig. 4B). This brings the increase in pleuromutilin production in *A. oryzae* to 2,106% over *C. passeckerianus*. We also estimated the titre of 14-*O*-acetyl-mutilin **2,** produced by the *three A. oryzae* transformants. Strain NSAR1 7 TR27 produced the highest titre of 74.52 mg/l, strain NSAR1 7 TR52 produced 18.33 mg/l, whereas no detectable amounts of **2** were observed in NSAR1 7 TR51, the highest producer of pleuromutilin **1** (Fig. 4B).

We carried out Southern blot analysis to detect copy number of the transgenes in these three same transformant strains and detected the presence of multiple copies of the genes *Pl-atf* and *Pl-sdr* in the highest producing strain NSAR1 7 TR51 (Supplementary Table 4). Multiple copies of the genes for the three cytochrome P450 s were found in strain NSAR1 7 TR52 (titre of **1:** 15.67 mg/l). This does not appear to provide any further increase in antibiotic titre compared to strain NSAR1 7 TR27 (titre of **1:** 21.86 mg/l) which has a single copy for each transgene.

## Discussion

The emergence of antibiotic resistance is posing a major concern for human health, and the development of robust alternatives to the currently-exploited antimicrobial agents has therefore become of primary importance. As the newest class of antibiotics for use in human therapeutics, and a class with great potential to treat various resistant and highly virulent pathogens such as Methicillin-resistant *Staphylococcus aureus* (MRSA) and Multi-Drug Resistant (MDR)-tuberculosis, the pleuromutilin antibiotics are becoming increasingly important. Nevertheless, the genetic basis of pleuromutilin production has not before been elucidated. For this reason we set out to identify the pleuromutilin gene cluster in *C. passeckerianus*, which is currently used to commercially produce pleuromutilin – and investigate potential approaches to increase pleuromutilin yields.

We hypothesized that, pleuromutilin being a diterpene compound, a pleuromutilin gene cluster may contain a pathway specific geranylgeranyl diphosphate synthase gene (*ggs*), as is the case for many characterized fungal diterpene gene clusters^16^,^17^,^18^. By screening a λ-phage genomic DNA library of *C. passeckerianus* we isolated four *ggs* genes. Of these four genes, only *ggs-2* was novel, the other three having been previously cloned and sequenced^19^. *ggs-2* was also the only ggs shown to be highly expressed under pleuromutilin production conditions, and was shown to be present only once in the genome, making it highly likely that this was the *ggs* responsible for providing geranyl-geranyl diphosphate (GGPP) for the biosynthesis of pleuromutilin and referred to thereafter as *Pl-ggs*.

Genome walking and sequencing of other λ-phage clones allowed the identification of genes adjacent to *Pl-ggs* with potential roles in secondary metabolite biosynthesis. These included a gene encoding a cyclase (*Pl-cyc*), which commonly catalyzes the cyclization of GGPP to give the first cyclic intermediate found in the biosynthesis of diterpenes, as well as genes encoding an acetyl transferase (*Pl-atf*), a short-chain dehydrogenase/reductase (*Pl-sdr*), three cytochrome P450s (*Pl-p450*-*1*, *Pl-p450-2* and *Pl- p450-3*), a zinc-binding dehydrogenase (*Cp-zbdh*), a flavin-binding monooxygenase-like protein (*Cp-fbm*), and four other putative proteins (*Cp-pp1*, *Cp-pp2*, *Cp-at* and *Cp-epl1*). Differential expression of the putative pleuromutilin gene cluster (*Pl-ggs*, *Pl-cyc*, *Pl-atf*, *Pl-sdr*, *Pl-p450-1*, *Pl-p450-2* and *Pl-p450-3*), under pleuromutilin production and non-production conditions was assessed through Northern blot analysis. Quantitative PCR analysis confirmed that these seven genes had high expression under production conditions of pleuromutilin, as well as being tightly co-regulated. This allowed the borders of the putative gene cluster to be defined.

By employing the molecular tools that we previously developed for genetic manipulation of *C. passeckerianus*^12^, we attempted to increase antibiotic production titres through overexpression of genes from the putative pleuromutilin gene cluster. Lines of the fungus with additional copies of each of the genes *Pl-ggs*, *Pl-cyc*, *Pl-atf*, *Pl-sdr*, *Pl-p450-1*, *Pl-p450-2*, *Pl-p450-3* and *Cp-fbm* under control of their native promoter sequence, were generated. Rather than leading to an increase in pleuromutilin titres, in multiple transformed strains, there was a reduction or loss of pleuromutilin production. This points to *C. passeckerianus* having a ‘sense-suppression’ mechanism, whereby the introduction of an exogenous copy of a gene will silence both the exogenous and endogenous copies. Such a phenomenon has been seen in other basidiomycete fungi such as *Cryptococcus neoformans*^20^ and *Schizophyllum commune*^21^.

Overexpression of six selected genes (*Cp-fds*, *Pl-ggs*, *Pl-cyc*, *Pl-p450-1*, *Pl-p450-2* and *Pl-p450-3*) was also attempted by placing them under the control of the *gpdII* promoter of *Agaricus bisporus*, which is known to drive strong expression in *C. passeckerianus*^12^. Although not present in the pleuromutilin gene cluster, the farnesyl diphosphate synthase gene (*Cp-fds*) was included within this set as increasing substrate availability to a pathway can increase production. Only one strain overexpressing *Pl-ggs* showed approximately 50% increase in pleuromutilin titre, whereas all other strains showed either no change in pleuromutilin production, or a reduction in titres. The whole genomic region containing the putative cluster was also cloned and introduced in additional copies to the genome of *C. passeckerianus*, but this also failed to provide a consistent and reproducible significant increase in antibiotic production.

Since the manipulation of the pleuromutilin gene cluster in the native host did not achieve a robust increase in antibiotic titre, total biosynthesis of pleuromutilin was recreated in a secondary host, with the aim of providing an alternative platform for improved production of the antibiotic. The ascomycete fungus *Aspergillus oryzae* was used for this purpose, as it has been used before as recipient organism for total biosynthesis of other fungal SMs, such as the diterpene aphidicolin^22^ and the polyketide tenellin^23^. The cDNA sequences of the seven genes identified as constituting the gene cluster in *C. passeckerianus* – *Pl-ggs*, *Pl-cyc*, *Pl-atf*, *Pl-sdr*, *Pl-p450-1*, *Pl-p450-2* and *Pl-p450-3* – were cloned and placed under control of constitutive promoters for expression in *A. oryzae*, using a modified version of the expression vectors designed by Pahirulzaman, Williams and Lazarus^12^. *De novo* production of pleuromutilin in *A. oryzae* transformants was assessed via plate-based bioassay, as well as purification and consequent NMR characterization of novel compounds. The metabolite 14-O-acetyl-mutilin, described in literature as a side-product from fermentation of pleuromutilin-producing fungi^14^, could also be isolated from the same strains. Quantification of pleuromutilin demonstrated that the *A. oryzae* transformants were producing higher pleuromutilin titres than *C. passeckerianus*, with one strain giving a remarkable 10-fold increase, which increases to 20-fold if the total fermentation time required is taken into account. Moreover, further improvement of the pleuromutilin-producing *A. oryzae* strain can now be achieved through classical means of random mutagenesis in a well understood host, as well as by promoting conversion of the putative precursor 14-*O*-acetyl-mutilin into pleuromutilin. Notably, among the pleuromutilin-producing *A. oryzae* strains tested, the strain that showed highest production of pleuromutilin also exhibited no detectable amounts of 14-*O*-acetyl-mutilin, whereas one strain with lower pleuromutilin production showed higher amounts of 14-*O*-acetyl-mutilin. A higher rate of conversion from 14-*O*-acetyl-mutilin to pleuromutilin could be achieved through improved expression of the gene responsible for catalyzing the putative last step of the pathway. The use of a well-known host such as *A. oryzae*, which produces few other secondary metabolites will also increase the efficiency of downstream purification.

The work described here has identified and defined the gene cluster for the important diterpene antibiotic pleuromutilin, from the basidiomycete fungus *C. passeckerianus*. The lack of consistent enhancement in antibiotic titre obtained through overexpression of the gene cluster in the native host suggests that, despite the availability of molecular tools, *C. passeckerianus* is not amenable to genetic manipulation aimed at improving metabolite production. Ultimately, seven clustered genes – *Pl-ggs*, *Pl-cyc*, *Pl-atf*, *Pl-sdr*, *Pl-p450-1*, *Pl-p450-2* and *Pl-p450-3* – were identified and confirmed to be sufficient to ensure biosynthesis of pleuromutilin, as shown by *de novo* production of the antibiotic through heterologous expression in *A. oryzae*. This also established a potential alternative method for production of the antibiotic in high titres, which will be needed to support large-scale production of pleuromutilin as novel semi-systemic pleuromutilin derivatives are launched.

Beyond the importance of this work to the field of pleuromutilin research, this also represents the first successful heterologous expression of an entire basidiomycete secondary metabolite gene cluster in a well-known ascomycete host. This demonstrates the potential value of this approach for producing natural products from the under exploited and often intractable basidiomycete fungi, in titres which are suitable for commercial exploitation.

## Materials and Methods

### Bacterial and yeast strains

The *Escherichia coli* strains routinely used for this study, JM109 and KW251 (both Promega, Southampton, UK), were grown on Luria-Bertani (LB) agar plates, supplemented when necessary with either ampicillin (100 μg/ml) or tetracycline (15 μg/ml). *Bacillus subtilis* was grown on Tryptic Soy Agar (TSA). *E. coli* One Shot^®^ ccdB Survival^TM^ 2 T1^R^ competent cells (Life Technologies) were used for the propagation of any plasmids containing a gateway cassette.

*Saccharomyces cerevisiae* BY4742 (genotype MATα, his3Δ1, leu2Δ0, lys2Δ0, ura3Δ0)^24^, was used for homologous recombination-based construction of plasmids, and was maintained on YPDA plates (10 g L^−1^ yeast extract, 20 g L^−1^ bactopeptone, 20 g L^−1^ D-glucose, 15 g L^−1^ agar) at 28 °C.

### Fungal strains and growth conditions

*Aspergillus oryzae* strain NSAR1 (genotype niaD^−^, sC^−^, ΔargB, adeA^−^)^25^, was used as heterologous host. This strain was maintained at 28 °C on MEA plates with appropriate supplements (15 g L^−1^ malt extract, 1.5 g L^−1^ arginine, 1.5 g L^−1^ methionine, 0.1 g L^−1^ adenine, 2 g L^−1^ ammonium sulphate, 15 g L^−1^ agar).

*Clitopilus passeckerianus* strain ATCC 34646 was grown routinely on potato dextrose agar (PDA; Sigma, UK) at 25 ^o^C for five days. For isolation of genomic DNA four 1 cm^2^ plugs of mycelial mat were used to inoculate 100 ml of CSO1A media (4 ml/L rape seed oil, 50 g/L glucose, 12.5 g/L yeast extract, 1.0 g/L KH_2_PO_4_, 0.5 g/L MgSO_4_⋅ 7H_2_O, 0.7 g/L Ca(N0_3_)2.4H_2_O, 0.1 g/L NaCl, 0.05 g/L FeSO_4_⋅ 7H_2_O; pH 6.2) and grown at 25 ^o^C for two days with agitation at 230 rpm. Following growth, the mycelium was collected by filtration through Miracloth (Calbiochem Corporation, La Jolla, Calif.), washed with TSE buffer (150 mM NaCl, 100 mM EDTA, 50 mM Tris-HCl pH 8.0), and blotted dry. Samples were ground in liquid nitrogen and used for extraction of genomic DNA.

For the preparation of RNA, *C. passeckerianus* was grown in 50 ml of PVS seed medium (8 g/L rape seed oil, 35 g/L spray dried corn liquor, 15 g/L glucose, 5 g/L calcium carbonate, pH 5.9) for three to five days at 25 ^o^C with agitation at 230 rpm. Two ml of the resulting seed media was used to inoculate production or non-production media. Production media (CGC) contained 50 g/L glucose, 5 g/L spray dried corn steep liquor and 2 g/L calcium carbonate, pH 6.5. Non-production media (MM) contained 50 mg/L adenine sulphate, 2 g/L L-asparagine, 25 ml/L of stock A [40 g/L KH_2_PO_4_, 90 g/L Na_2_HPO_4_, 11.6 g/L Na_2_SO_4_, 20 g/L Di-Ammonium tartrate], 1 ml/L of stock B [40 mg/L Thiamine], 10 ml/L of stock solution C [25 g/L MgSO_4_] and 10 g/L glucose, added after autoclaving, pH 6.5.

Cultures were harvested as described above for DNA extraction and stored at −80 ^o^C for the preparation of RNAs. Stock cultures of *C. passeckerianus* were maintained on potato dextrose agar at 4 ^o^C or as agar plugs in 20% glycerol at −80 ^o^C.

### Nucleic acid isolation

Genomic DNA was isolated from *C. passeckerianus* mycelia by the method of Porebski *et al.*^26^. Plasmid and lambda DNA were isolated using Qiagen (Qiagen Ltd, UK) kits. Total RNA was isolated using the RNA plant mini kit (Qiagen).

### Southern and Northern Blots

For Southern blots, genomic DNA was digested to completion overnight with a suitable range of restriction enzymes (New England Biolabs), and separated by agarose gel electrophoresis. For Northern blots total RNA (10 μg) was separated on a 1.2% formaldehyde agarose gel. Genomic DNA digests and total RNA were transferred to positively charged nylon membrane (Amersham Life Science) using a vacuum blotter (Bio-rad, UK). Transferred RNA was fixed to membranes by UVP CL-1000 UV cross-linker (Genetic Research Instrumentation Ltd, Essex, England). Filters were probed with [α-32P] (3,000 Ci/mmol; Amersham, Buckinghamshire, United Kingdom). The labelling of DNAs was performed using Ready-To-Go™ DNA Labelling Beads (Amersham Pharmacia Biotech). Unincorporated [α-^32^P]dCTP was removed using ProbeQuant G-50 microspin columns (Amersham Pharmacia Biotech). Hybridization with 32P-labelled probes were carried out at 65 ^o^C overnight in Church Buffer (1% BSA, 1 mM EDTA, 0.5 M NaPO4 pH 7.2, 7% SDS). The membranes were rinsed in 2 x SSC (20 x SSC per L: 175.3 g NaCl, 88.2 g sodium citrate) at room temperature, followed by a 20 min wash at 65 ^o^C in 2 x SSC and then a wash in 0.5 x SSC at 65 ^o^C for 20 min. Damp filters were placed on Kodak X-Omat AR autoradioagraphy film (Sigma) and allowed to expose for the desired time with intensification at −80 ^o^C to detect hybridization signals.

### Degenerate PCR

PCR products amplified with GoTaq DNA polymerase (Promega) were purified with Wizard SV Gel (Promega) and PCR Clean-Up System (Promega). These products were routinely cloned into pGEM-T easy (Promega) and transformed into *E. coli* JM109. PCRs were carried out in a final volume of 20 μl containing 1 × Green Go-Taq PCR buffer (Promega), 0.4 mM of each primer, 100 μM of dNTPs, 2.5 mM MgCl_2_ and 1U of Go-Taq DNA polymerase (Promega) and 5 ng of DNA. Reaction conditions for PCR amplification consisted of an initial denaturation step at 94 ^o^C for 2 min followed by 30 cycles of: denaturation at 94 ^o^C for 30 sec, annealing at 50–65 ^o^C for 30 sec and extension at 72 ^o^C for 1 min per kb, with a final extension of 10 min at 72 ^o^C. Reactions were carried out in a MJ Research PTC 200 Thermal Cycler (MJ Research, Watertown, MA). Degenerate primers fdsf2A and fdsr4 were designed by using conserved sequences identified from an alignment of *Cryptococcus neoformans* var. *neoformans* JEC21(AAW43830), *Lactarius chrysorrheus* (BAD15361) and *Ustilago maydis* 521 (XP_757593) and used to amplify a region of *Cp-fds* gene. To generate a *ggs* PCR product, degenerate primers pair ggs27 and ggs29, originally designed by Zhang *et al.*^27^ were used.

### Real-Time PCR

Maxima SYBR Green/ROX qPCR Master Mix (Fermentas) was used for all reactions (Maxima SYBR Green/ROX qPCR Master Mix: 12.5 μl, Forward primer: 0.3 μM, Reverse primer: 0.3 μM, Template cDNA: 25–50 ng, Water to 25 μl). Reaction conditions were 10 minutes at 95 °C followed by 40 x [95 °C for 15 secs, 60 °C for 30 secs, 72 °C for 30 secs]. This was followed by one cycle of 95 °C for 1 min, 60 °C for 30 secs and 95 °C for 30 secs to calculate the disassociation curves for each reaction. This was used to confirm that the desired amplified product is being detected rather than primer dimers, contamination or misannealed primer product. All reactions were carried out using the Mx3005P^TM^ qPCR instrument (Stratagene).

To analyse the results MX-PRO software, which is part of the Mx3005P^TM^ qPCR system, was used. The threshold fluorescence was set using the amplification-based algorithm according to the software manufacturer’s instructions. The Ct values for each reaction were normalised using β-tubulin as the reference gene. Samples were then compared to a calibrator sample (either wild-type or non-production) to gain relative quantification of transcript levels.

### Construction and screening of a *C. passeckerianus* genomic library

Fifty micrograms of DNA were partially digested with *Sau*3AI to generate the maximum yield of fragments in the size range 9–23 kb. The DNA was then size-fractionated and cloned in *Bam* HI-linearized vector Lambda GEM-11 (Promega) following the vector’s instruction manual. *In-vitro* packaging was performed using the Packagene Lambda DNA System (Promega). Propagation and amplification of the genomic library were performed by infection of *E. coli* KW251. Aliquots of the amplified library were stored in 7% DMSO at −80 ^o^C. The library was screened by plaque hybridization with *C. passeckerianus ggs* and *fds* probes using standard methods^28^. To confirm number and position of introns in gene sequences, 3′ RACE (Rapid Amplification of cDNA Ends) was performed using the Ambion FirstChoice RLM RACE kit.

### DNA sequencing and Bioinformatics

Sequencing reactions, in both directions, were carried out by GlaxoSmithKline (Harlow, United Kingdom) and Agowa (Berlin, Germany). Sequence data was assembled into contigs using Sequencher, version 4.7 (Gene Codes). Database similarity searches were performed using the National Centre for Biotechnological Information (NCBI) online program BLAST (http://www.ncbi.nlm.nih.gov/BLAST)^29^. The PROSITE database was used to identify motifs and signature sequences in the deduced protein sequences with homology to reported proteins (http://www.expasy.ch/tools/scanprosite/)^30^. Sequence data were aligned using the ClustalW program (http://www.ebi.ac.uk/clustalw/)^31^. DNA and protein sequences were analysed using the Sequence Manipulation website (http://www.ualberta.ca/~stothard/javascript/)^32^.

### Construction of *C. passeckerianus* plasmids

Vectors with the selectable marker genes *hph* (hygromycin resistance) and *cbx* (carboxin resistance) were constructed through yeast-based homologous recombination^33^. The *cbx* cassettes were amplified from pCbx004 and pCbxi004^12^ using chimeric primers yeast_AgaricusgpdII_promf and yeast_AspergillustrpC_termr (Supplementary Table 1), then individually recombined into a 5.8 kb *Xba*I-*Hind*III fragment of yeast shuttle vector pYES2 (Invitrogen, UK), along with the 1.9 kb fragment of *hph* cassette, which was amplified from pPHT1 by using chimeric primers yeast_coprinustub_promf and yeast_coprinustub_termr (Supplementary Table 1). This resulted in the plasmids pYES-hph-cbxgene and pYES-hph-icbx respectively (Supplementary Figure 6A). The plasmids are identical except the 64 bp intron-exon region of *A. bisporus gpdII* gene in pYES-hph-icbx. These plasmids were used as bench plasmids to replace either selectable marker with genes of interest and transform *C. passeckerianus*.

To produce *Pl-cyc* antisense silencing cassette, the coding regions of *Pl-cyc* was amplified in their entirety with suitable primer tails to allow recombination into a *Xho*I and *Bam*HI fragment of pYES-hph-cbxgene, placing the gene in the antisense orientation under the control of the *A. bisporus gpdII* promoter.

To produce *Pl-cyc* antisense silencing cassette, the coding regions of *Pl-ggs* was amplified in their entirety with suitable primer tails to allow ligation into a *Nco*I and *Bam*HI fragment of pCbx004^12^, placing the gene in the antisense orientation under the control of the *A. bisporus gpdII* promoter.

In order to clone the pleuromutilin pathway genes under the control of their native promoter sequences, individual coding regions of genes with their promoter sequences were amplified from the lambda clones of a genomic library of *C. passeckerianus*, using corresponding chimeric primers with 30 bp-overlap with the pYES2 vector sequence (Supplementary Table 1). These were individually recombined into a *Xho*I and *Bam*HI fragment of pYES-hph-cbxgene, resulting in overexpression vectors containing pleuromutilin pathway genes under the control of their native promoters and *A. nidulans trpC* terminator sequences (Supplementary Figure 6B).

To obtain plasmids with the pleuromutilin pathway genes under the control of the *A. bisporus gpdII* promoter and *A. nidulans trpC* terminator, the coding regions of genes were amplified from the corresponding lambda clones of *C. passeckerianus* genomic library, using respective chimeric primers with 30 bp-overlaps with *A. bisporus gpdII* promoter and *A. nidulans trpC* terminator sequence (Supplementary Table 1). For genes *Pl-cyc*, *Pl-p450-1*, *Pl-p450-2* and *Pl-p450-3* the coding region were individually recombined into a *Xho*I and *Bam*HI fragment of plasmid pYES-hph-cbxgene, resulting in overexpression vectors containing pleuromutilin pathway genes under the control of *A. bisporus gpdII* promoter and *A. nidulans trpC* terminator sequences (Supplementary Figure 6C). In order to verify the influence of an intron on gene expression levels, the coding regions were also amplified using a chimeric forward primer with homology to the 64 bp intron-exon region of *A. bisporus gpdII*. This allowed recombination into pYES-hph-icbxgene, placing the coding regions downstream of a 5′ intron. For genes *Cp-fds* and *Pl-ggs*, the coding regions were individually ligated into *Nco*I and *Bam*HI fragments of pcbx004 and pcbxi004^12^.

To clone the entire pleuromutilin gene cluster of 25 kb (consisting of coding regions of nine putative genes: *Pl-p450-3*, *Pl-atf*, *Pl-cyc*, *Pl-ggs*, *Pl-p450-1*, *Pl-p450-2*, *Pl-sdr*, *Cp-zbdh* and *Cp-fbm*) under the control of their native regulatory sequences, the whole 25 kb cluster sequence was amplified as five 5 kb fragments. Each fragment was amplified from the corresponding lambda clones, allowing at least 100 bp-overlaps between adjacent fragments. Fragment 1 was amplified from λ42, fragment 2 from λ34, fragment 3 from λG4, fragments 4 and 5 were amplified from λ5. All 5 fragments were recombined into a *Xho*I and *Bam*HI fragment of plasmid pYES-hph-cbx by yeast-based homologous recombination resulting in pYES-hph-pleurocluster (Supplementary Figure 6D).

### PEG-mediated transformation of *C. passeckerianus*

Recombinant plasmids were transformed into *C. passeckerianus* protoplasts as described by Kilaru *et al.*^12^.

### Bio-assay to determine the pleuromutilin production levels

To determine the pleuromutilin production levels, *C. passeckerianus* transformants and wild type strain were analysed by bio-assay as described in Hartley *et al.*^1^. This technique was also employed for initial screening of *A. oryzae* transformants for antibacterial activity. Wild-type *A. oryzae* and *C. passeckerianus* were used as negative and positive controls respectively.

### Heterologous production of pleuromutilin

Expression vectors for the heterologous expression of pleuromutilin were constructed through homologous recombination in yeast following the published procedure^33^. The seven genes were amplified from cDNA using Phusion^®^ High-Fidelity DNA Polymerase with extended primers (Supplementary Table 1). Three expression vectors, pTYGSargGGSCyc, pTYGSadeP450s and pTYGSbarATFSDR (Fig. 3A), were constructed based on those developed by Pahirulzaman, Williams and Lazarus^34^ and were used to transform *A. oryzae* NSAR1. Protoplast-mediated transformation of *A. oryzae* was carried out following the published protocol^35^.

### Screening *A. oryzae* transformants for expression and production

*Aspergillus oryzae* transformants were analysed for expression of the transgenes and production of new metabolites. Each transformant strain was grown in 100 mL of CMP medium (35 g L^−1^ Czapek-dox liquid, 20 g L^−1^ maltose, 10 g L^−1^ peptone) at 28 °C for five days prior to proceeding with RNA extraction and RT-PCR for expression analysis. A ten-day culture of each transformant grown under the same conditions was subject to extraction in ethyl acetate, concentrated *in vacuo* and dissolved in methanol. The crude extract was analysed by HPLC for detection of new metabolites and preparative HPLC was used to purify novel metabolites.

## Additional Information

**How to cite this article**: Bailey, A. M. *et al.* Identification and manipulation of the pleuromutilin gene cluster from *Clitopilus passeckerianus* for increased rapid antibiotic production. *Sci. Rep.* **6**, 25202; doi: 10.1038/srep25202 (2016).

## Supplementary Material

Supplementary Information

## Figures and Tables

**Table 1:** Genes sequenced during this project.

	Gene	Length (bp)	Number of introns	Predicted protein length (aa)	Putative function	Closest known homologue and accession number
FDS Locus	*Cp-fds*	1487	5	375	Farnesyl diphosphate synthase (FDS)	FPPS from *Lactarius chrysorrheus* (BAD15361)
	*Cp-cdb*	3436	21	764	Cellobiose dehydrogenase	*cbd* from *Coniophora puteana* (BAD32781)
	*Cp-ATPase*	4549	14	1225	Cation transporter	Cation transporting ATPase from *Aspergillus fumigatus* Af293 (XP_754245)
	*Cp-ccp*	435	0	144	Cerato-platanin protein	cerato-platanin 6 from *Crinipellis campanella* (AGL40521)
Pleuromutilin Locus	*Pl-p450-3*	2108	10	522	Cytochrome P450	*Pleurotus sapidus* putative Cytochrome P450 monooxygenase (CAJ00405)
	*Pl-atf*	1304	3	377	Acetyltransferase	*Aspergillus fumigatus* Acetyltransferase TRI7-like toxin biosynthesis protein (XP_746970)
	*Pl-cyc*	3040	3	959	Terpene Cyclase	*Gibberella fujikuroi* Ent-kaurene synthase (Ent-copalyl diphosphate synthase) (Q9UVY5)
	*Pl-ggs*	1291	4	350	Geranylgeranyl pyrophosphate synthetase (GGS)	*Mucor racemosus* Geranylgeranyl pyrophosphate synthetase (Q9P885)
	*Pl-p450-1*	2286	13	523	P450 monooxygenase	*Pleurotus sapidus* cytochrome P450-3 monooxygenase (CAL69751)
	*Pl-p450-2*	2124	11	525	P450 monooxygenase	*Pleurotus sapidus* cytochrome P450-3 monooxygenase (CAL69751)
	*Pl-sdr*	945	3	254	Short-chain dehydrogenase/reductase (SDR)	*Desulfitobacterium hafniense* DCB-2 short-chain dehydrogenase/reductase SDR (ZP_01373200)
	*Cp-zbdh*	1479	10	321	Oxidoreductase	*Neosartorya fischeri* NRRL 181 oxidoreductase, zinc-binding dehydrogenase family (XP_001264189)
	*Cp-fbm*	2434	11	616	Monooxygenase	*Aspergillus fumigatus* Af293 flavin-binding monooxygenase-like protein (XP_751903)
	*Cp-pp1*	2144	21	338	Unknown	Predicted protein from *Laccaria bicolor* S238N-H82 (XP_001884957)
	*Cp-pp2*	–	–	–	Unknown	predicted protein from *Coprinopsis cinerea* (EAU81003).
	*Cp-at (partial)*	–	–	–	Putative aminotransferase	*Moniliophthora roreri* aminotransferase family protein (XP_007868896)

**Table 2:** Transformants obtained with vectors containing pleuromutilin pathway genes under the control of their native promoters.

Gene Expressed	Plasmid	Total transformants	Transformants assayed	Clearing zone diameter range (% of wild-type)	Average clearing zone diameter (% of wild-type)
*p450-3*	pYES-hph-nativep450-3gene	221	50	80–113%	98.4%
*atf*	pYES-hph-nativeATFgene	188	50	76–122%	106.0%
*cyc*	pYES-hph-nativeCycgene	189	56	0–128%	96.9%
*ggs*	pYES-hph-nativeGGSgene	179	45	0–121%	102.8%
*p450-1*	pYES-hph-nativep450-1gene	167	56	0–142%	111.1%
*p450-2*	pYES-hph-nativep450-2gene	156	53	60–122%	104.2%
*sdr*	pYES-hph-nativeSDRgene	98	50	84–112%	98%
*fbm*	pYES-hph-nativeFBMgene	138	52	80–113%	98.4%

A number of each set were analysed by bioassay to estimate pleuromutilin production. The range and average clearing zone diameters are shown as a percentage of wild-type *C. passeckerianus*.

**Table 3:** Total number of transformants obtained with vectors containing pleuromutilin pathway genes under the control of *A. bisporus gpdII* promoter.

Gene Expressed	Plasmid	Total transformants	Pleuromutilin titre range (μg/g)	Clearing zone range (and average) as a percentage of wild-type
*FDS*	p004-CP1-FDSgene	55	595–810	–
*FDS*	p004-CP1-iFDSgene	20	600–920	–
*GGS*	p004-CP1-GGSgene	32	495–1,010	–
*GGS*	p004-CP1-iGGSgene	22	605–795	–
*Cyclase*	pYES-hph-Cycgene	31	–	0–109.4% (89.0%)
*Cyclase*	pYES-hph-iCycgene	23	–	100.8–122.0% (106.7%)
*P450-1*	pYES-hph-p450-1gene	20	–	70.4–103.0% (92.4%)
*P450-1*	pYES-hph-ip450-1gene	23	–	77.0–103.7% (92.5%)
*P450-2*	pYES-hph- p450-2gene	22	–	68.1–107.3% (89.3%)
*P450-2*	pYES-hph-ip450-2gene	30	–	83.7–105.2% (94.2%)
*P450-3*	pYES-hph-ip450-3gene	32	–	49.7–113.8% (98.3%)

The FDS and GGS lines were analysed by HPLC. All others were analysed by bioassay. The titres of pleuromutilin recorded through HPLC (μg of pleuromutilin per g of dry mycelial mass) are reported for the transformant lines overexpressing the genes *fds* and *ggs*. The wild-type levels were 690–815 μg/g.
