# Supplementary material for: Identification and manipulation of the pleuromutilin gene cluster from Clitopilus passeckerianus for increased rapid antibiotic production
Source: Sci Rep. 2016 May 4;6:25202. doi: 10.1038/srep25202 (PMC4855138; doi:10.1038/srep25202)
Supplement: Supplementary Information [file srep25202-s1.pdf]

Identification and manipulation of the pleuromutilin gene cluster

from *Clitopilus passeckerianus* for increased rapid antibiotic production

Andy M. Bailey[1], Fabrizio Alberti[1], Sreedhar Kilaru[1], Catherine M. Collins[1], Kate de Mattos-Shipley[1], Amanda J. Hartley[1], Patrick Hayes[1], Alison Griffin[2], Colin M. Lazarus[1], Russell J. Cox[3], Christine L. Willis[3], Karen O'Dwyer[4], David W. Spence[3] and Gary D. Foster[1] *

1. School of Biological Sciences, Life Sciences Building, University of Bristol, 24 Tyndall Avenue, Bristol, BS8 1TQ, UK

2. GSK, Southdownview Way, Worthing, West Sussex, BN14 8QH, United Kingdom

3. School of Chemistry, University of Bristol, Cantock's Close Bristol, BS8 1TS, UK

4. GSK, 1250 S. Collegeville Road, Collegeville, Pennsylvania, 19426-0989, United States

* Author for Correspondence

Contributions

GDF, AMB, DS and K O'D devised the initial project. GDF and AMB lead the Bristol based team; DWS and K O'D at GSK. CLW and RJC contributed to the organic chemistry. CML contributed to design of Aspergillus strategy. AMB, FA, K de M-S, SK, CMC, AJH, PH & AG carried out the experiments and analysis, it should be noted that AMB, FA, SK, CMC, K de M-S all contributed equally, and author position does not signify any particular role or seniority. AMB supported all computational and chemical analysis. K de M-S and FA compiled the first drafts of the manuscript, with final version written by GDF with contributions from other members of the team.

Supplementary data

Supplementary Figure 1: Northern blots for three *ggs* genes under pleuromutilin production conditions. *ggs-2* was the only ggs highly expressed under production conditions. The *ggs-2* film was exposed for 4 hours. In comparison, it was necessary to expose the films for *ggs-3* and *ggs-4* for 24 hours and 3 days respectively, to achieve a signal.

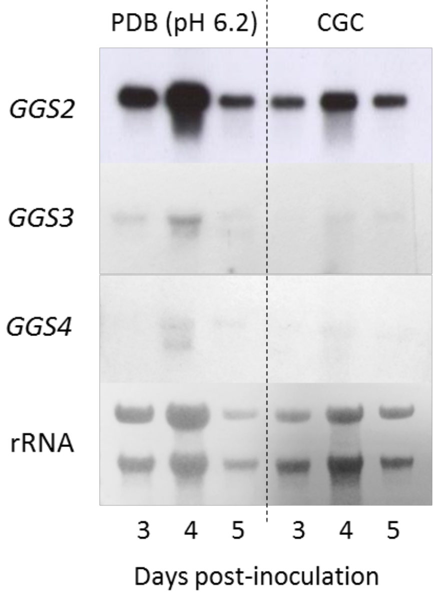

Supplementary Figure 2: Southern analyses of *ggs-2 (Pl-ggs)* (**A**) and *cp-fds* (**B**) genes. Restriction enzymes used: *Pst*I, *Sal*I, *Xho*I, Undigested genomic DNA. The presence of two bands in some lanes is due to the dikaryotic nature of *C. passeckerianus.*

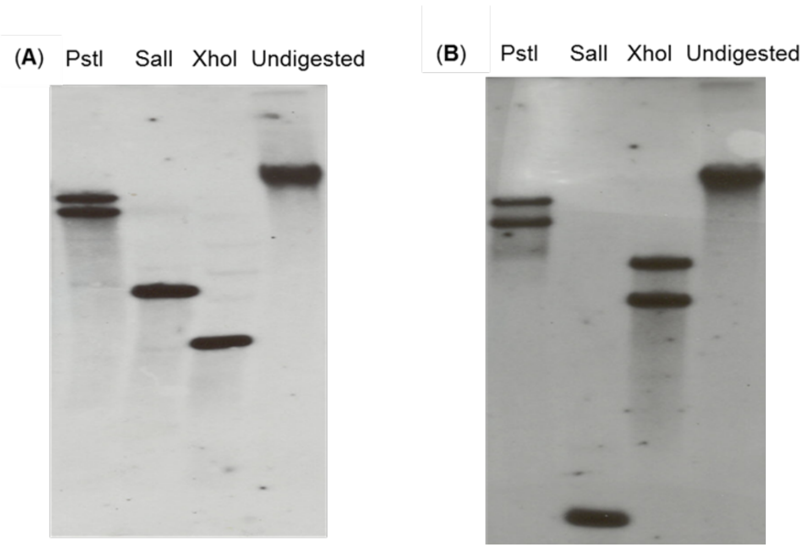

Supplementary Table 1. Primers sequences. Primers were designed based on the sequence data generated for *C. passeckerianus* ATCC 34646. Italicized bases are homologous to vectors or other DNA fragments so that the amplified product can be used downstream in yeast-based homologous recombination. The non-italicized bases in these primers represent the bases homologous to the target gene.

Primer	Sequence (5'->3')	Description
**fdsf2A**	CTACTCCTTCTATCTCCCTGTCGC	Amplification of *Cp-fds* through degenerate PCR
**fdsr4**	CCAVGAGCAYTTRTTGTC	
**fds_Start_NcoI**	*CCCCC*ATGGCCTCCGATAAAGCTGCACG	Amplification of *Cp-fds* gene
**fds_end_BamHI**	*GGGGATCC*TCATTTGCTTCGGCCGTAGATC	
**ggs27**	CAYMGIGGTCARGGTATGGA	Amplification of *Pl-ggs* through degenerate PCR
**ggs29**	AACTTTCCYTCIGTSARGTCYTC	
**p450-3_promoter_f**	*ACGGATTAGAAGCCGCCGAGCGGGTGACAG*GGAAGTACAGGAATGTCAAGAC	Amplification of *Pl-p450-3* promoter
**p450-3_promoter_r**	*GGTCGGGCTGTGTGGTGTACTGACCGCCAT*AGATGTTGGGAAGACTCTCG	
**P450-3_start**	*CTCCCATCTACACACAACAAGCTTATCGCC*ATGGCTCCGTCAACGGAACGTG	Amplification of *Pl-p450-3* gene
**P450-3_end**	*TTTGATGATTTCAGTAACGTTAAGTGGATC*CTAGCCACTAGCAGGCTTCGTG	
**p450-3_intron_start**	*CGACCGCGTGCTGACTTCGCTTTCCAGGCC*ATGGCTCCGTCAACGGAACGTG	Amplification of *Pl-p450-3* gene with 30bp overlap to an intron
**P450-3 FF**	cccaacatctatggctccgtcaacg	Amplification of *Pl-p450-3* with UTRs
**P450-3 RR**	acatgtgtatctagccactagcagg	
**P450-3 START FF**	ATGGCTCCGTCAACGGAACGTGCTC	Amplification of *Pl-p450-3* coding sequence
**P450-3 STOP RR**	CTAGCCACTAGCAGGCTTCGTGAAC	
**Peno-P450-3 FF**	*GTCGACTGACCAATTCCGCAGCTCGTCAAA* ATGGCTCCGTCAACGGAACG	Amplification of *Pl-p450-3* to be used in yeast homologous recombination
**P450-3-Teno RR**	*GGTTGGCTGGTAGACGTCATATAATCATAC* CTAGCCACTAGCAGGCTTCG	
**ATF_promoter_f**	*ACGGATTAGAAGCCGCCGAGCGGGTGACAG*TATGGCTAGTTCGGTAGAATATAC	Amplification of *Pl-atf* promoter
**ATF_promoter_r**	*GGTCGGGCTGTGTGGTGTACTGACCGCCAT*GGTGGTATCAGTCCAAGGAGG	
**ATF_start**	*CTCCCATCTACACACAACAAGCTTATCGCC*ATGAAGCCCTTCTCACCA	Amplification of *Pl-atf* gene

	GA	
**ATF_end**	*TTTGATGATTTCAGTAACGTTAAGTGGATC*TCATGTATTGCCGCCGCCAT	
**ATF FF**	tgataccaccatgaagcccttctca	Amplification of *Pl-atf* with UTRs
**ATF RR**	acgggcgtgcctactgtgctacacg	
**ATF START FF**	ATGAAGCCCTTCTCACCAGAACTTC	Amplification of *atf* coding sequence
**ATF STOP RR**	CTACTGTGCTACACGAGGGGGATTC	
**Padh-ATF FF**	*TTTCTTTCAACACAAGATCCCAAAGTCAAA* ATGAAGCCCTTCTCACCAGA	Amplification of *Pl-atf* to be used in yeast homologous recombination
**ATF-TgpdA RR**	*ACGACAATGTCCATATCATCAATCATGACCCTA*CTGTGCTACACGAGGGG	
**ATF Southern RR**	GTTAGAGCCAGACTTCAAGCCTAGA	Amplification of probe for *Pl-atf* to be used in Southern blot
**Cyclase_promoter_f**	*ACGGATTAGAAGCCGCCGAGCGGGTGACAG*CGTATGAATTTAGGGAGGTAA	Amplification of *Pl-cyc* promoter
**Cyclase_promoter_r**	*GGTCGGGCTGTGTGGTGTACTGACCGCCAT*AGTGAAGATGAGAGCGCAGC	
**Cyclase_start**	*CTCCCATCTACACACAACAAGCTTATCGCC*ATGGGTCTATCTGAAGATCT	Amplification of *Pl-cyc* gene
**Cylcase_end**	*TTTGATGATTTCAGTAACGTTAAGTGGATC*CTAATAAACACAATCATTCATGT	
**Cyclase_intron_start**	*CGACCGCGTGCTGACTTCGCTTTCCAGGCC*ATGGGTCTATCTGAAGATCT	Amplification of *Pl-cyc* gene with 30bp overlap to an intron
**Cyclase_antisense_f**	*TTTGATGATTTCAGTAACGTTAAGTGGATC* ATGGGTCTATCTGAAGATCT	Amplification of *cyc* gene to be used in antisense construct
**Cyclase_antisense_r**	*CTCCCATCTACACACAACAAGCTTATCGCC*CTAGCGATATCAATGGTGGA	
**Cyclase FF**	ctctcatcttcactatgggtctatc	Amplification of *Pl-cyc* with UTRs
**Cyclase RR**	actagccatatcaatggtggattcc	
**Cyclase START FF**	ATGGGTCTATCTGAAGATCTTCATG	Amplification of *Pl-cyc* coding sequence
**Cyclase STOP RR**	TCAATGGTGGATTCCATTGCTCCCG	
**CYCLASE internal FF1**	CCACTCACGACGCTGACATGAGCTC	Internal sequencing of *Pl-cyc*
**CYCLASE internal RR1**	ACCTCGCTGAGGGTCGAGAACGACT	
**Peno-CYCLASE FF**	*GTCGACTGACCAATTCCGCAGCTCGTCAAA* ATGGGTCTATCTGAAGATCT	Amplification of *Pl-cyc* to be used in yeast homologous recombination
**CYCLASE-Teno RR**	*GGTTGGCTGGTAGACGTCATATAATCATACTCA*ATGGTGGATTCCATTG	

	C	
**Cyc Southern RR**	AGCAGGCGATGGACACCCTGGACAG	Amplification of probe for *Pl-cyc* to be used in Southern blot
**GGS_Start_BspHI**	*CCCTC*ATGAGAATACCTAACCCGGTC	Amplification of *Pl-ggs* gene
**GGS_end_NcoI**	*GGGGGATCC*CTACTCTGCGATGTACAAC	
**GGS_promoter_f**	*ACGGATTAGAAGCCGCCGAGCGGGTGACAG*AGTGAAGATGAGAGCGCAGC	Amplification of *Pl-ggs* promoter-gene
**GGS_end**	*TTTGATGATTTCAGTAACGTTAAGTGGATC*CTACTCTGCGATGTACAACTT	
**GGSstart_BamHI**	*CCCGGATCC*ATGAGAATACCTAACGTC	Amplification of *ggs* gene to be used in antisense construct
**GGSend_NcoI**	*GGGCCATGG*CTACTCTGCGATGTACAAC	
**GGS FF**	aattcatacgatgagaatacctaac	Amplification of *Pl-ggs* with UTRs
**GGS RR**	agattcttatctactctgcgatgta	
**GGS START FF**	ATGAGAATACCTAACGTCTTTCTCT	Amplification of *Pl-ggs* coding sequence
**GGS STOP RR**	CTACTCTGCGATGTACAACTTTTCC	
**Padh-GGS FF**	*TTTCTTTCAACACAAGATCCCAAAGTCAAA*ATGAGAATACCTAACGTCTT	Amplification of *Pl-ggs* to be used in yeast homologous recombination
**GGS-TgpdA RR**	*ACGACAATGTCCATATCATCAATCATGACCCTA*CTCTGCGATGTACAACT	
**p450-1_promoter_f**	ACGGATTAGAAGCCGCCGAGCGGGTGACAG*ATAGGGGGATTGCCGACGTT*	Amplification of *Pl-p450-1* promoter
**p450-1_promoter_r**	GGTCGGGCTGTGTGGTGTACTGACCGCCAT*TGCGTGAATAAAGCTCGAGC*	
**p450-1_start**	*CTCCCATCTACACACAACAAGCTTATCGCC*ATGCTGTCCGTCGACCTC	Amplification of *Pl-p450-1* gene
**p450-1_complete_end**	*TTTGATGATTTCAGTAACGTTAAGTGGATC*CTACAACGCAGCGAACGCT	
**p450-1_intron_start**	*CGACCGCGTGCTGACTTCGCTTTCCAGGCC*ATGCTGTCCGTCGACCTC	Amplification of *Pl-p450-1* gene with 30bp overlap to an intron
**P450-1 FF**	tattcacgcaatgctgtccgtcgac	Amplification of *Pl-p450-1* with UTRs
**P450-1 RR**	tgttaggaggctacaacgcagcgaa	
**P450-1 START FF**	ATGCTGTCCGTCGACCTCCCGTCTG	Amplification of *Pl-p450-1* coding sequence
**P450-1 STOP RR**	CTACAACGCAGCGAACGCTTCCTTA	
**Padh-P450-1 FF**	*TTTCTTTCAACACAAGATCCCAAAGTCAAA*ATGCTGTCCGTCGACCTCCC	Amplification of *Pl-p450-1* to be used in yeast homologous recombination
**P450-1-Tadh RR**	TTCATTCTATGCGTTATGAACATGTTCCCTCTACAACGCAGCGAAC	

	GCTT	
**p450-2_promoter_f**	*ACGGATTAGAAGCCGCCGAGCGGGTGACAG*GGTCACTGCTGCGTATTGC	Amplification of *Pl-p450-2* promoter
**p450-2_promoter_r**	*GGTCGGGCTGTGTGGTGTACTGACCGCCAT*AGTCGTCCGAAAGTCTAGAC	
**p450-2_start**	*CTCCCATCTACACACAACAAGCTATCGCC*ATGAATCTTTCTGCTCTGAAGG	Amplification of *Pl-p450-2* gene
**p450-2_end**	*TTTGATGATTTCAGTAACGTTAAGTGGATC*CTAATAGTCTGCAACATCGTG	
**p450-2_intron_start**	*CGACCGCGTGCTGACTTCGCTTTCCAGGCC*ATGAATCTTTCTGCTCTGAAGG	Amplification of *Pl-p450-2* gene with 30bp overlap to an intron
**P450-2 FF**	tcggacgactatgaatctttctgct	Amplification of *Pl-p450-2* with UTRs
**P450-2 RR**	atccccctatctaatagtctgcaac	
**P450-2 START FF**	ATGAATCTTTCTGCTCTGAAGGCTG	Amplification of *Pl-p450-2* coding sequence
**P450-2 STOP RR**	CTAATAGTCTGCAACATCGTGGATC	
**PgpdA-P450-2 FF**	*AACAGCTACCCCGCTTGAGCAGACATCACC*ATGAATCTTTCTGCTCTGAA	Amplification of *Pl-p450-2* to be used in yeast homologous recombination
**P450-2-TgpdA RR**	*ACGACAATGTCCATATCATCAATCATGACC*CTAATAGTCTGCAACATCGT	
**SDR_promoter_f**	*ACGGATTAGAAGCCGCCGAGCGGGTGACAG*AGTCGTCCGAAAGTCTAGAC	Amplification of *Pl-sdr* promoter
**SDR_promoter_r**	*GGTCGGGCTGTGTGGTGTACTGACCGCCAT*GGTCACTGCTGCGTATTGCT	
**SDR_start**	*CTCCCATCTACACACAACAAGCTATCGCC*ATGGAAGGCAAGGTCGTGCT	Amplification of *Pl-sdr* gene
**SDR_end**	*TTTGATGATTTCAGTAACGTTAAGTGGATC*CTAAATGACACTCCACCCGTT	
**SDR FF**	agcagtgaccatggaaggcaaggtc	Amplification of *Pl-sdr* with UTRs
**SDR RR**	atacggcgacctaaatgacactcca	
**SDR START FF**	ATGGAAGGCAAGGTCGCAATCGTCA	Amplification of *Pl-sdr* coding sequence
**SDR STOP RR**	CTAAATGACACTCCACCCGTTATCG	
**Peno-SDR FF**	*GTCGACTGACCAATTCCGCAGCTCGTCAAA*ATGGAAGGCAAGGTCGCAAT	Amplification of *Pl-sdr* to be used in yeast homologous recombination
**SDR-Teno RR**	*GGTTGGCTGGTAGACGTCATATAATCATACCTA*AATGACACTCCACCCGT	
**SDR Southern FF**	TGTGTCCGGCTCCAAGGACGCCTTT	Amplification of probe for *Pl-sdr* to be used in Southern blot

**FBM_promoter_f**	*ACGGATTAGAAGCCGCCGAGCGGGTGACAG*TGACATAGTATGACCTCTGAA	Amplification of *Cp-fbm* promoter-gene
**FBM_end**	*TTTGATGATTTCAGTAACGTTAAGTGGATC*TTAGTTGGGTAAAGTGGCAAC	
**pJET1.2 FF**	CGACTCACTATAGGGAGAGCGGC	Sequencing of inserts from pJET1.2
**pJET1.2 RR**	AAGAACATCGATTTTCCATGGCAG	
**beta-tub RIB40 FF**	CCAAGAACATGATGGCTGCT	Amplification and qPCR of *beta-tubulin* from *A. oryzae*
**beta-tub RIB40 RR**	CTTGAAGAGCTCCTGGATGG	
**Fragment1_f**	*ACGGATTAGAAGCCGCCGAGCGGGTGACAG*AGCTTCGTACTGGCCGATTC	Amplification of fragment 1 of pleuromutilin gene cluster
**Fragment1_r**	CTGTGGCATGGTTCGTCTAC	
**Fragment2_f**	CTCTACACGTGGCGACAGCA	Amplification of fragment 2 of pleuromutilin gene cluster
**Fragment2_r**	TTGGCACCGCGAATCCGACT	
**Fragment3_f**	CTTGAGAGCGACAAGGCAGG	Amplification of fragment 3 of pleuromutilin gene cluster
**Fragment3_r**	GAGCTCGACATTGGTGAAGG	
**Fragment4_f**	GCCACATCTTCGTCATGAGAT	Amplification of fragment 4 of pleuromutilin gene cluster
**Fragment4_r**	CACACATGGGGTGTTGGGAG	
**Fragment5_f**	CTATCTCGCCTTCATCATCG	Amplification of fragment 5 of pleuromutilin gene cluster
**Fragment5_r**	*ATGCCAGAATTCCATGCACAATCAGCAGAT*TGACATAGTATGACCTCTGAA	
**Yeast_AgaricusgpdII_promf**	*ACGGATTAGAAGCCGCCGAGCGGGTGACAG*GAAGAAGAATTCAGAGGTCCG	Amplification of *cbx* cassette
**Yeast_AspergillustrpC_termr**	*ATGCCAGAATTCCATGCACAATCAGCAGAT*TGGAGATGTGGAGTGGGCG	
**Yeast_Coprinustub_promf**	*GCAAATTAAAGCCTTCGAGCGTCCCAAAACCT*TTCATTTAAACGGCTTCACGG	Amplification of *hph* cassette
**Yeast_Coprinustub_termr**	*ATCTGCTGATTGTGCATGGAATTCTGGCAT*CAATATTCATCTCTCCATCGAA	

Supplementary Figure 3: A Northern blot analysis of genes present in the putative pleuromutilin gene cluster. Differential expression between production and non-production conditions suggests that the true cluster c ontains *Pl-ggs*, *Pl-cyc*, *Pl-p450-1*, *Pl-p450-2*, *Pl-atf*, *Pl-p450-3* and *Pl-sdr*, and that *Cp-fbm* represents the edge of the pleuromutilin gene cluster.

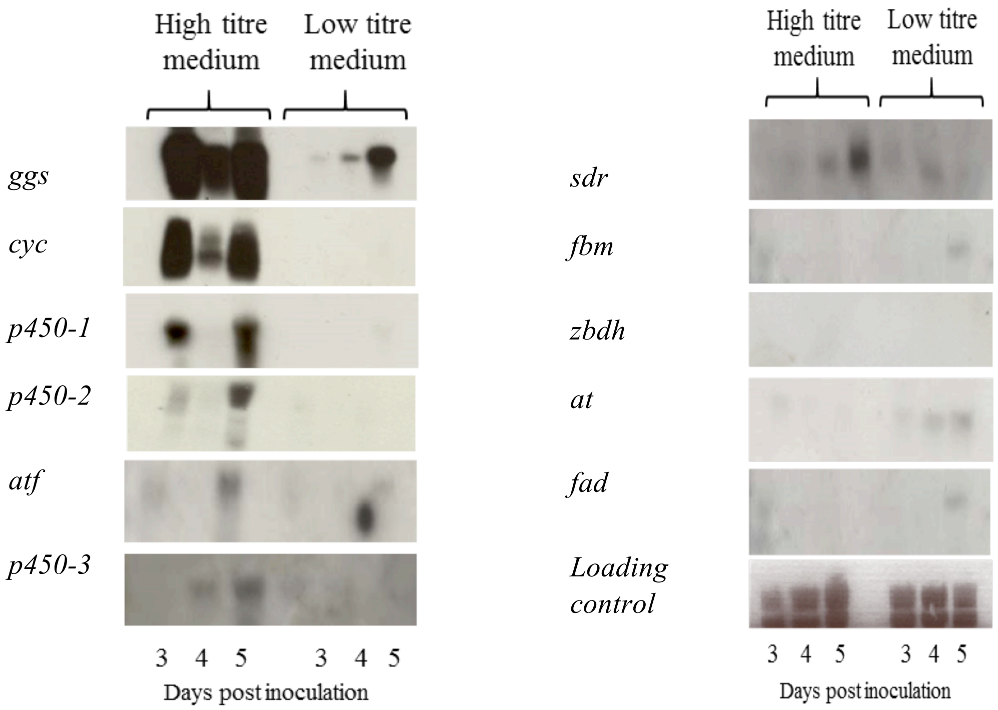

Supplementary Figure 4: A time-course for pleuromutilin production in *C. passeckerianus* when grown on CGC (a high-production medium) and MM (a non-production medium). Three flasks were analyzed for each day, with yields expressed as a percentage of maximum and standard deviation bars are shown.

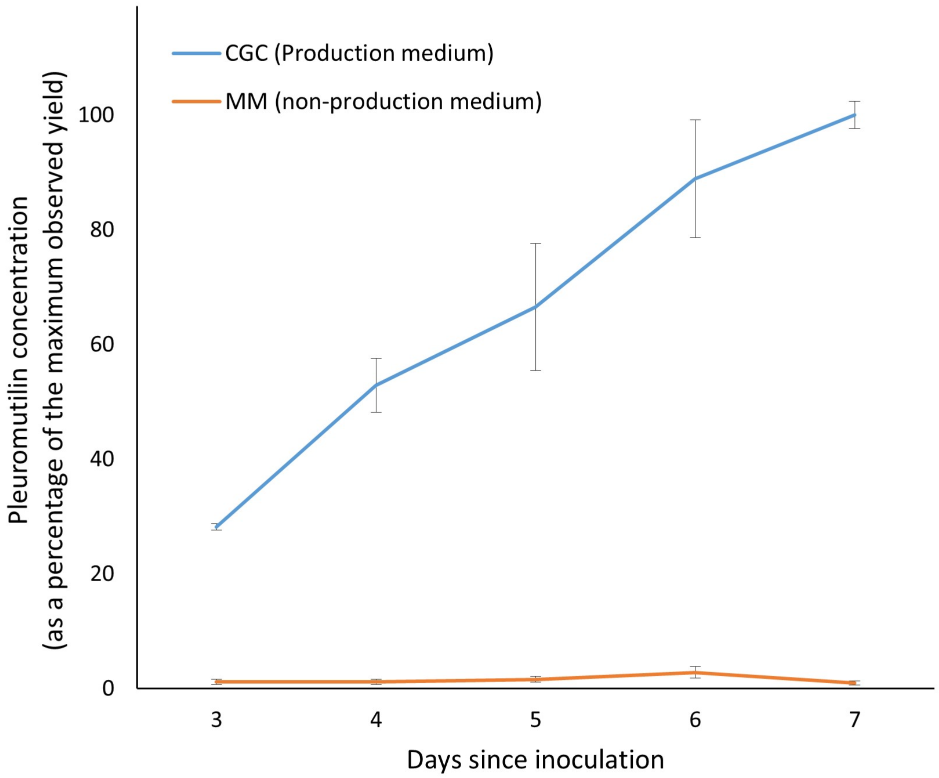

Supplementary Figure 5: An FDS (farnesyl diphosphate synthase) encoding gene (*Cp-fds*) was identified and sequenced. Adjacent genes have been identified as encoding a putative ATPase (*Cp-ATPase*), a cellobiose dehydrogenase (*Cp-cbd*) and a hypothetical protein. These genes are unlikely to belong to a secondary metabolite gene cluster.

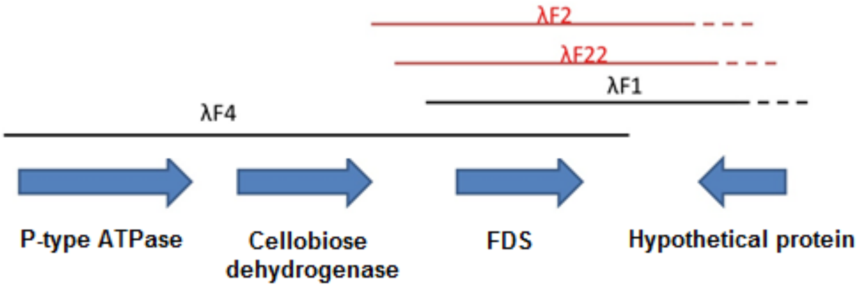

Supplementary Figure 6: A: Structure of plasmids pYES-hph-cbxgene and pYES-hph-icbxgene, which contain dual selectable marker genes. Hygromycin (*hph)* cassette, amplified from plasmid pPHT1[1], and carboxin (*cbx)* cassette, amplified from plasmid p004icbx[2], were cloned into yeast shuttle vector pYES2 (Invitrogen, UK) using yeast-based homologous recombination. *gpdII* promoter: glyceraldehyde-3-phosphate dehydrogenase promoter from *A. bisporus* was used to drive gene expression. A *gpdII* intron-exon region: 64 bp intron-exon region of *A. bisporus gpdII* gene was introduced only in pYES-hph-icbxgene. The *trpC* terminator: indole-3-glycerol phosphate synthase terminator from *A. nidulans* as well as *tub1* promoter and terminator: promoter and terminator from the tubulin gene of *Coprinus cinereus* were also used in plasmid construction. This plasmid was used as the backbone for producing the plasmids shown in B-D. B: General structure of plasmids pYES-hph-native_gene, containing individual genes from the pleuromutilin gene cluster (*Pl-p450-3*, *Pl-atf*, *Pl-cyc*, *Pl-ggs*, *Pl-p450-1*, *Pl-p450-2*, *Pl-sdr* or *Pl-fbm*) with their native promoter. C: General structure of plasmids pYES-hph-gene, containing individual genes from the pleuromutilin gene cluster controlled by the promoter sequence of *A. bisporus gpdII* gene (with or without 64 bp intron-exon region of *A. bisporus gpdII* at the 5' of the coding sequence of the gene). D: Structure of plasmid pYES-hph-pleurocluster. Here, the cbx cassette of pYES-hph-cbxgene has been replaced with 25 kb of the predicted pleuromutilin gene cluster.

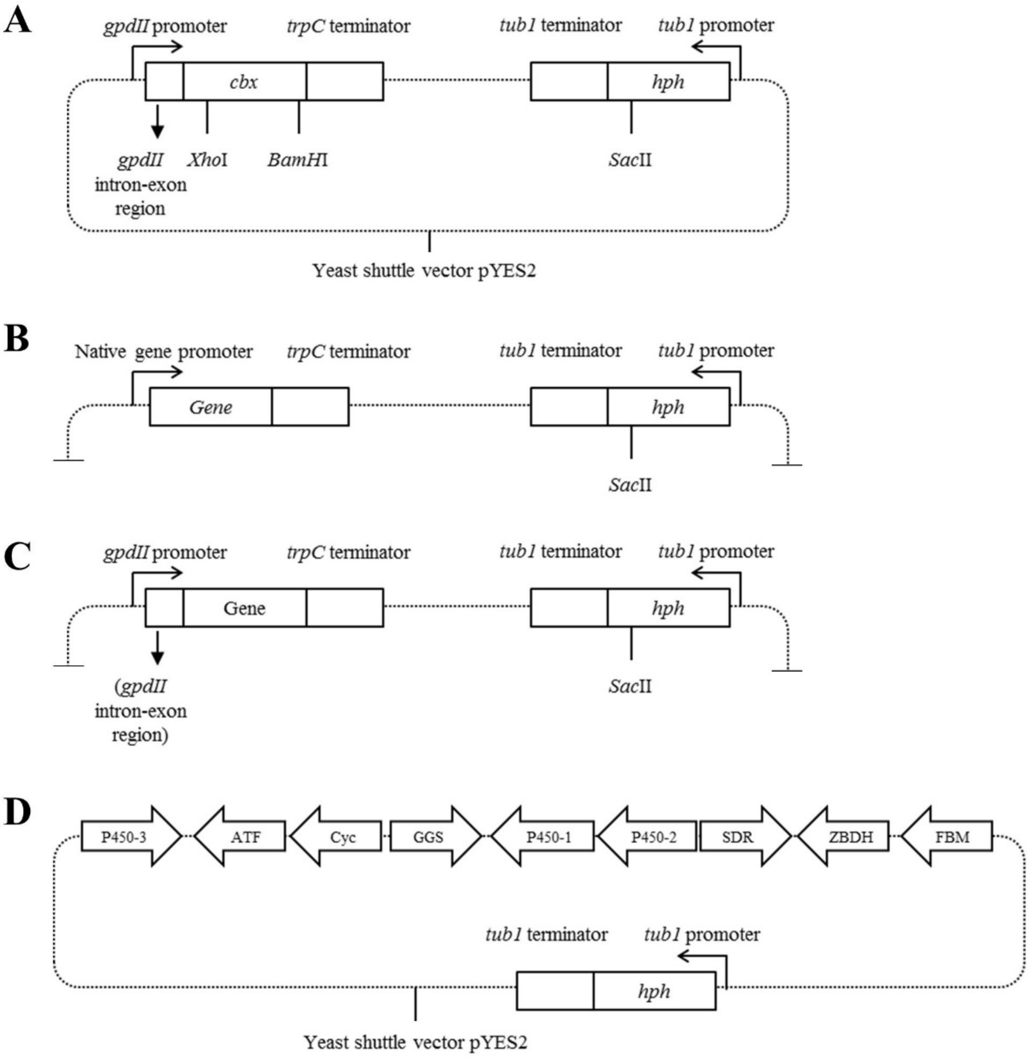
A
gpdII promoter
trpC terminator
tub1 terminator
tub1 promoter
cbx
hph
gpdII intron-exon region
XhoI
BamHI
SacII
Yeast shuttle vector pYES2
B
Native gene promoter
trpC terminator
tub1 terminator
tub1 promoter
Gene
hph
SacII
C
gpdII promoter
trpC terminator
tub1 terminator
tub1 promoter
Gene
hph
(gpdII intron-exon region)
SacII
D
P450-3
ATF
Cyc
GGS
P450-1
P450-2
SDR
ZBDH
FBM
tub1 terminator
tub1 promoter
hph
Yeast shuttle vector pYES2

Supplementary Figure 7: Antibacterial activities of silenced *C. passeckerianus* transformants on Tryptic Soy Agar (TSA). Clearing zones surrounding the fungal colony, due to an absence of *B. subtilis* growth, indicate the bactericidal action of pleuromutilin. **A**: Wild-type *C. passeckerianus*. **B**: A transformant containing the *Pl-ggs* silencing cassette. **C**: A transformant containing the *Pl-cyc* silencing cassette.

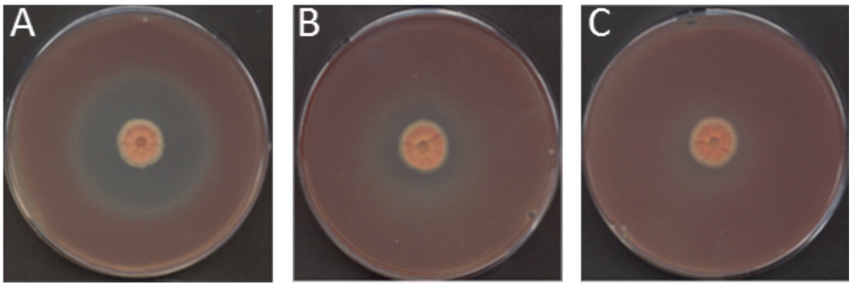

Supplementary Figure 8. Clearing zones recorded on plate-based bioassays for *C. passeckerianus* strains transformed with plasmid p004GGSantigene.

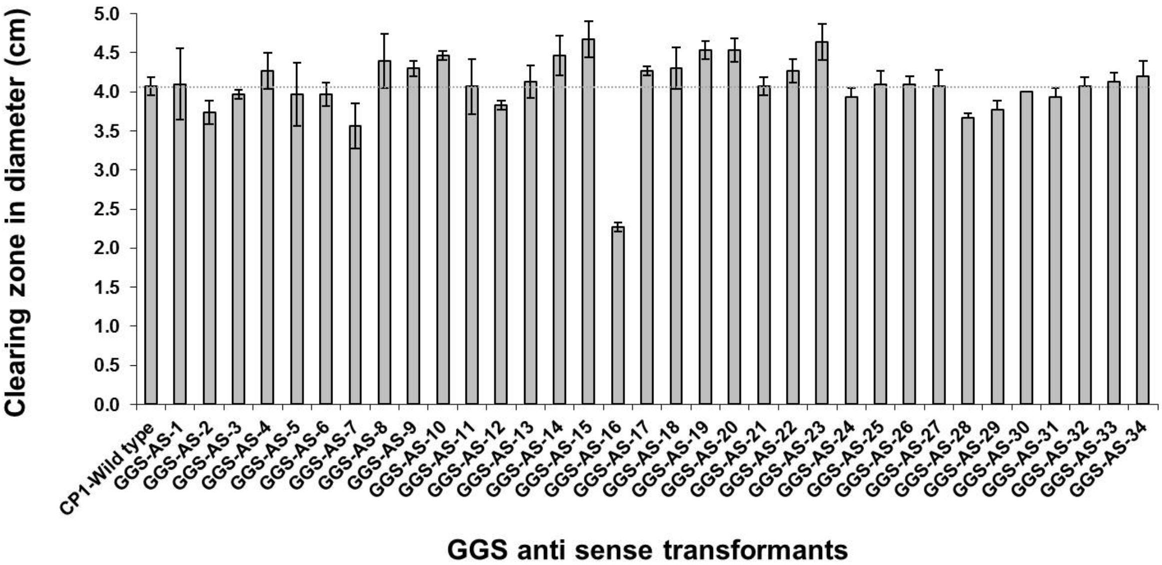

Supplementary Figure 9. Clearing zones recorded on plate-based bioassays for *C. passeckerianus* strains transformed with plasmid pYES-hph-CYCantigene

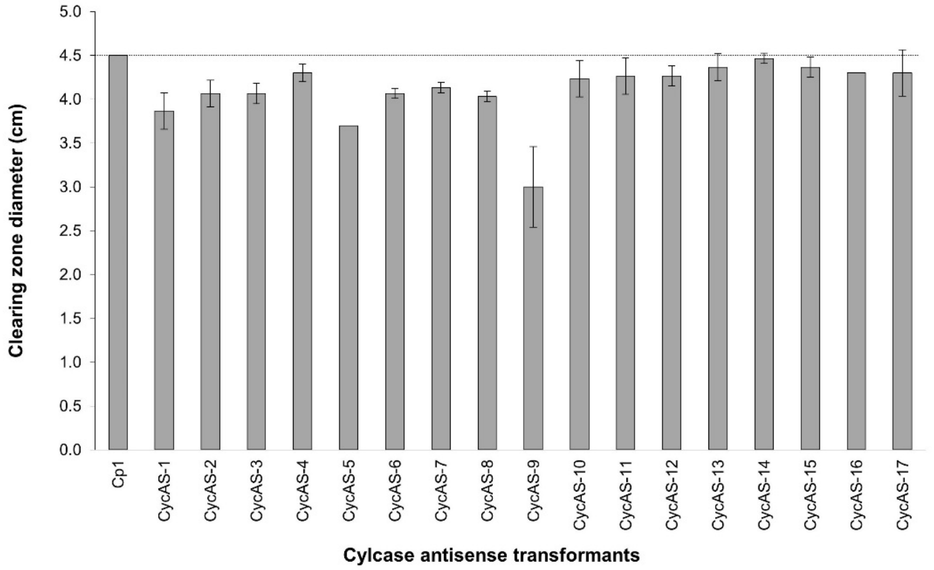

Supplementary Figure 10. Clearing zones recorded on plate-based bioassays for *C. passeckerianus* strains transformed with plasmid pYES-hph-nativeGGSgene.

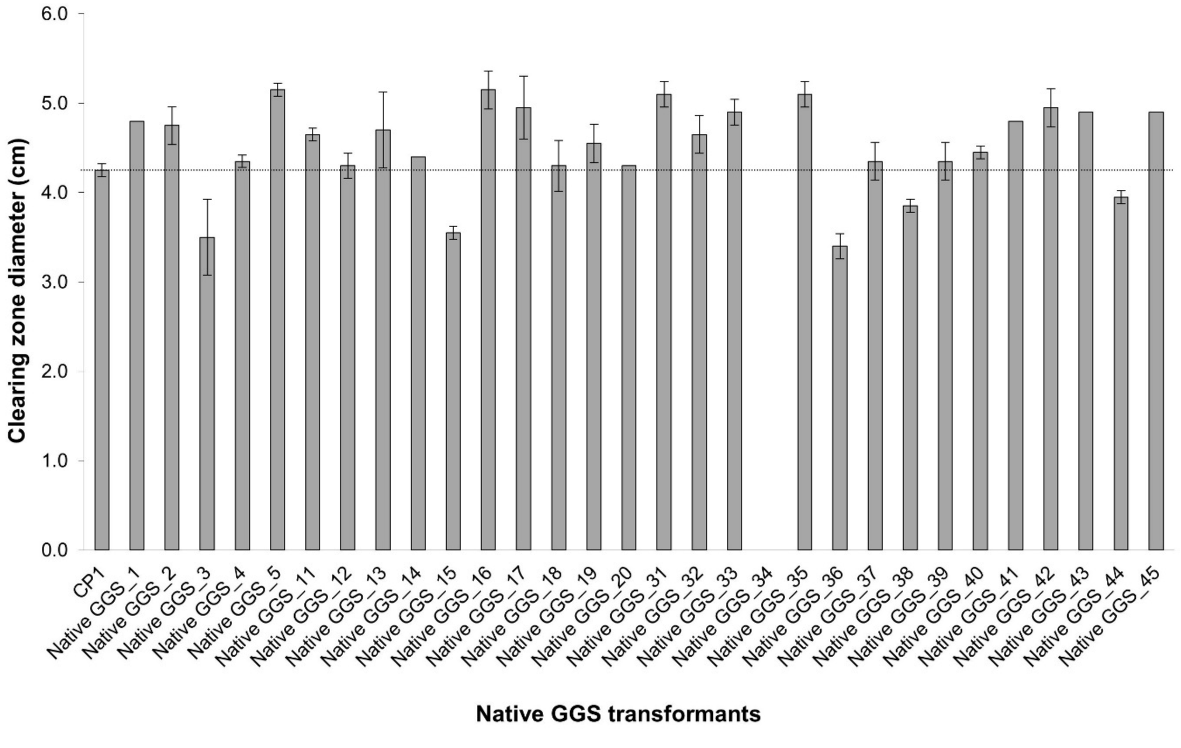

Supplementary Figure 11. Clearing zones recorded on plate-based bioassays for *C. passeckerianus* strains transformed with plasmid pYES-hph-nativeCycgene.

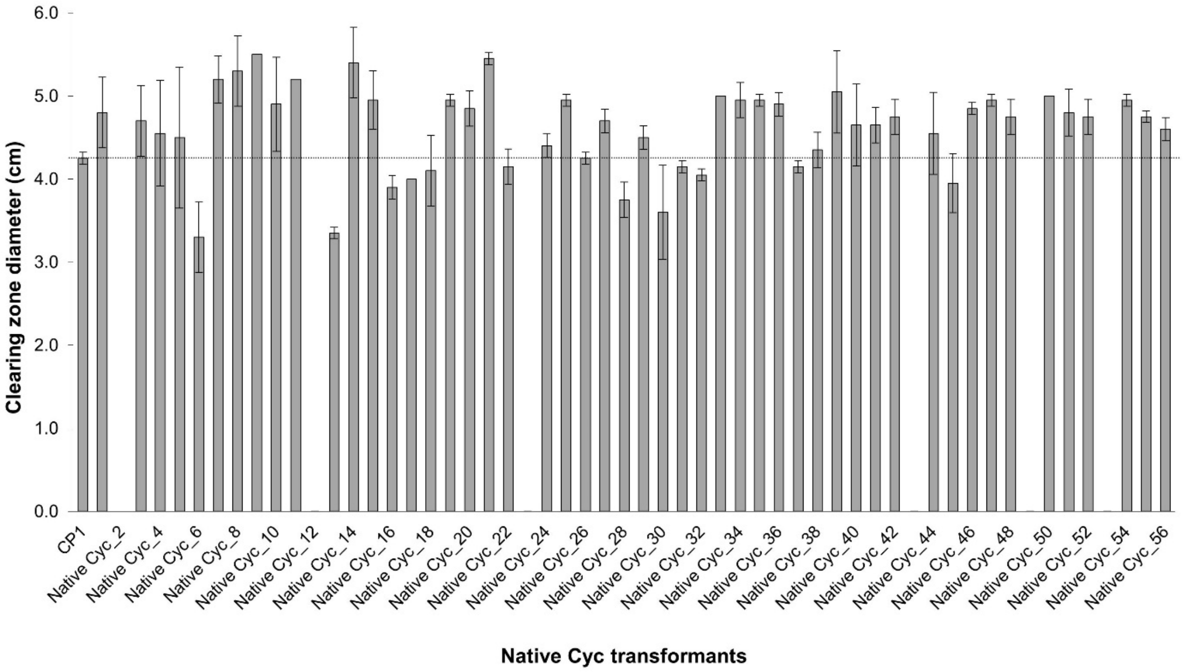

Supplementary Figure 12. Clearing zones recorded on plate-based bioassays for *C. passeckerianus* strains transformed with plasmid pYES-hph-nativep450-1gene.

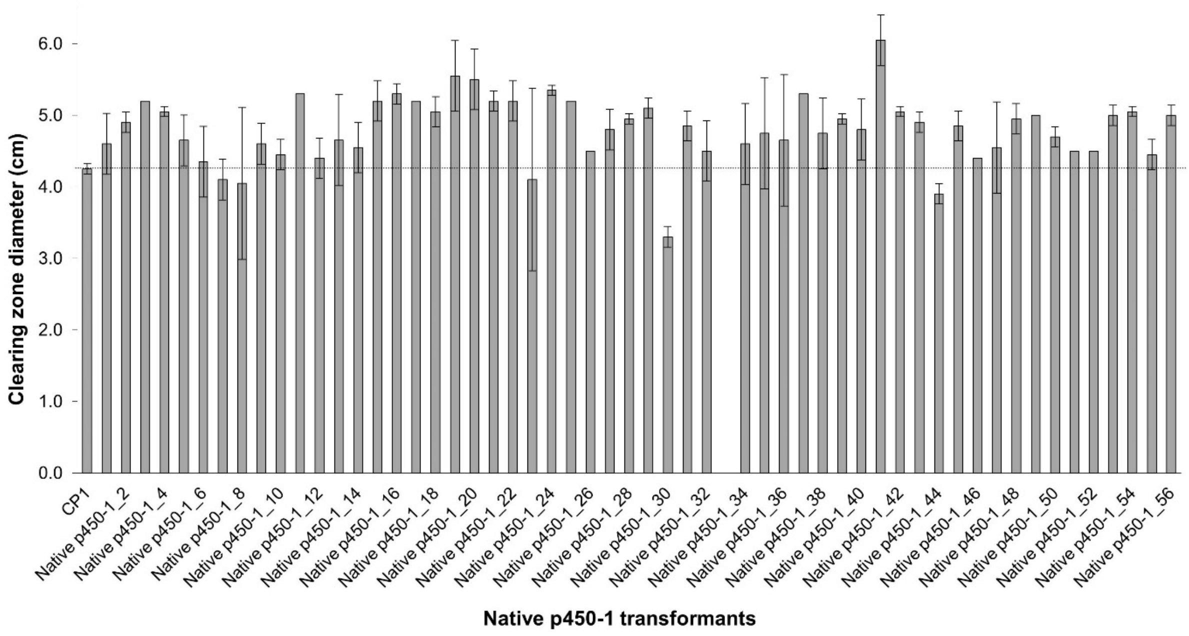

Supplementary Figure 13. Clearing zones recorded on plate-based bioassays for *C. passeckerianus* strains transformed with plasmid pYES-hph-nativep450-2gene.

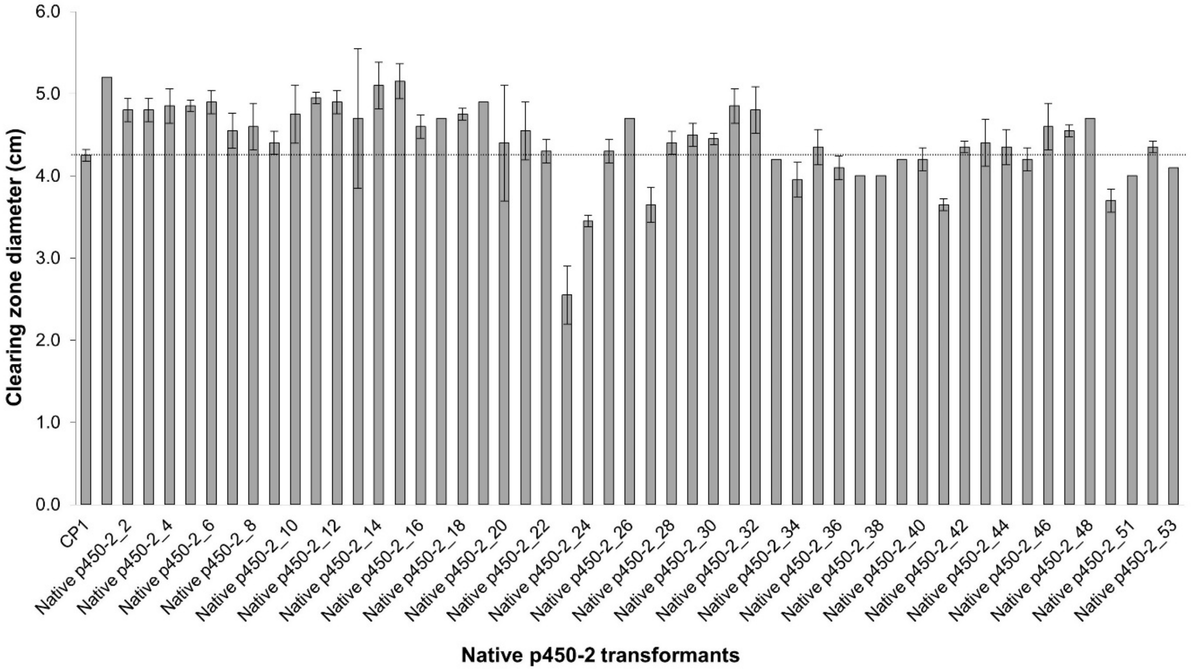

Supplementary Figure 14. Clearing zones recorded on plate-based bioassays for *C. passeckerianus* strains transformed with plasmid pYES-hph-nativep450-3gene.

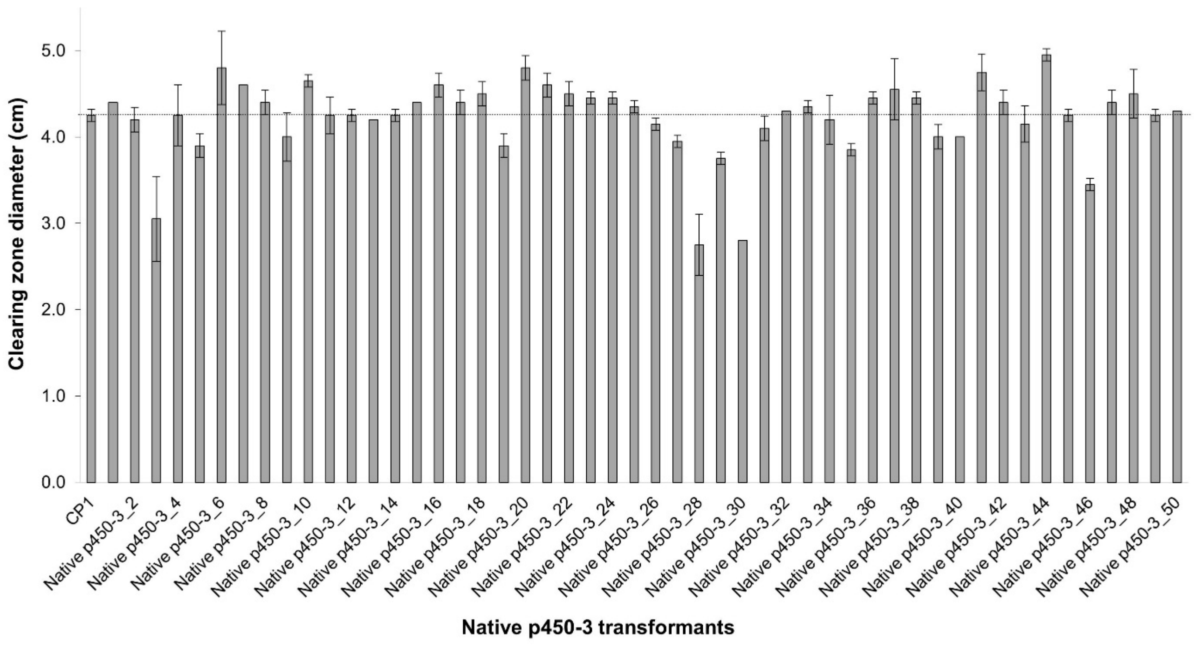

Supplementary Figure 15. Clearing zones recorded on plate-based bioassays for *C. passeckerianus* strains transformed with plasmid pYES-hph-nativeATFgene.

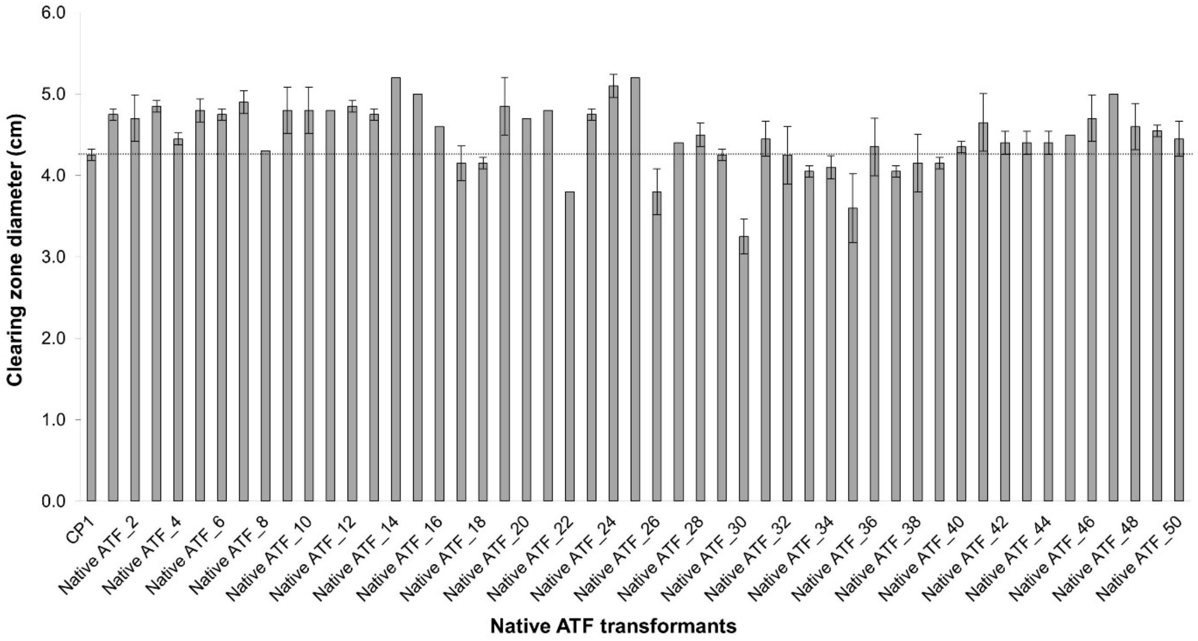

Supplementary Figure 16. Clearing zones recorded on plate-based bioassays for *C. passeckerianus* strains transformed with plasmid pYES-hph-nativeSDRgene.

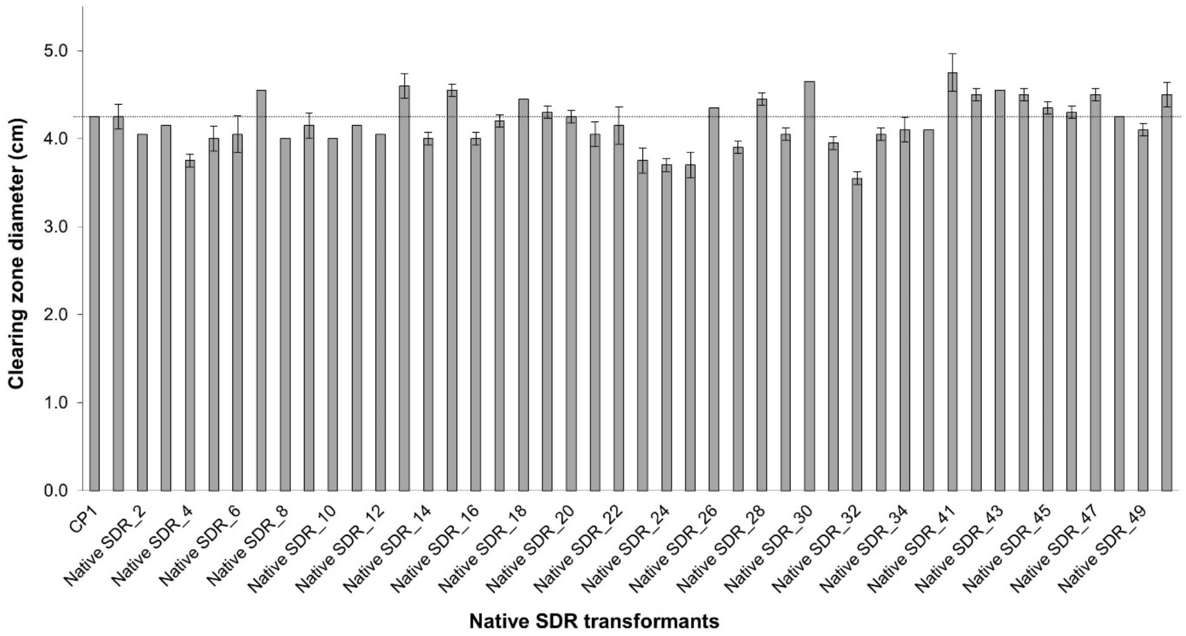

Supplementary Figure 17. Clearing zones recorded on plate-based bioassays for *C. passeckerianus* strains transformed with plasmid pYES-hph-nativeFBMgene.

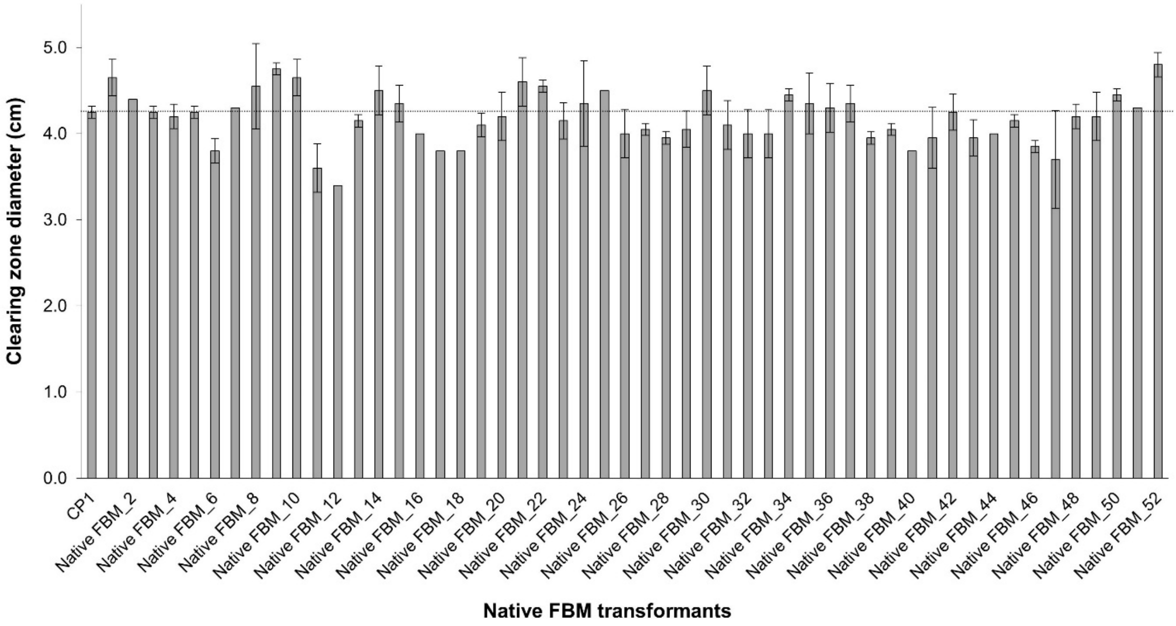

Supplementary Figure 18. Pleuromutilin titers (µg/g of mycelia) of *C. passeckerianus* wild type, *ggs* sense transformant 16 and antisense transformant 16.

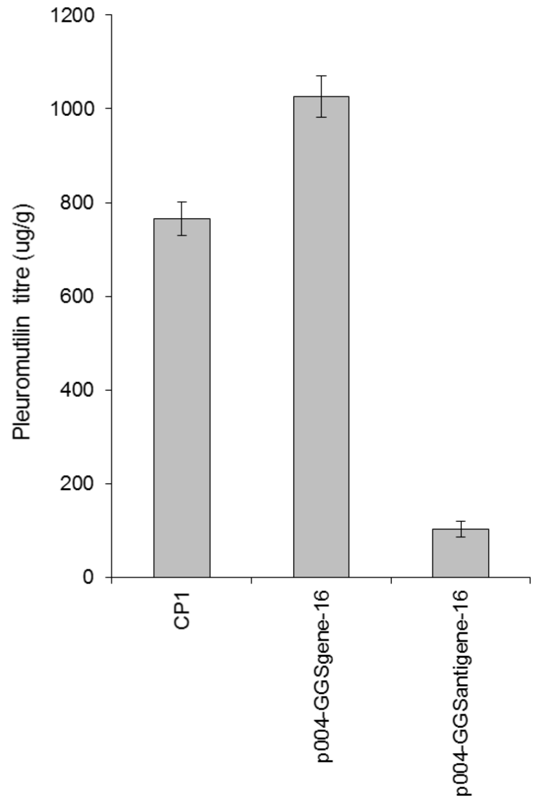

Supplementary Figure 19. Northern analysis of cultures obtained from pYES-hph-GGSgene transformant-16 (lane 1) and *C. passeckerianus* wild type (lane 2). (**A**) Total RNA stained with methylene blue (**B**) Blot was hybridized with *ggs* probe.

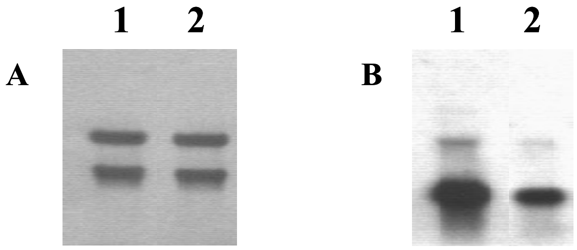

Supplementary Figure 20. Clearing zones recorded on plate-based bioassays for *C. passeckerianus* strains transformed with plasmid pYES-hph-Cycgene.

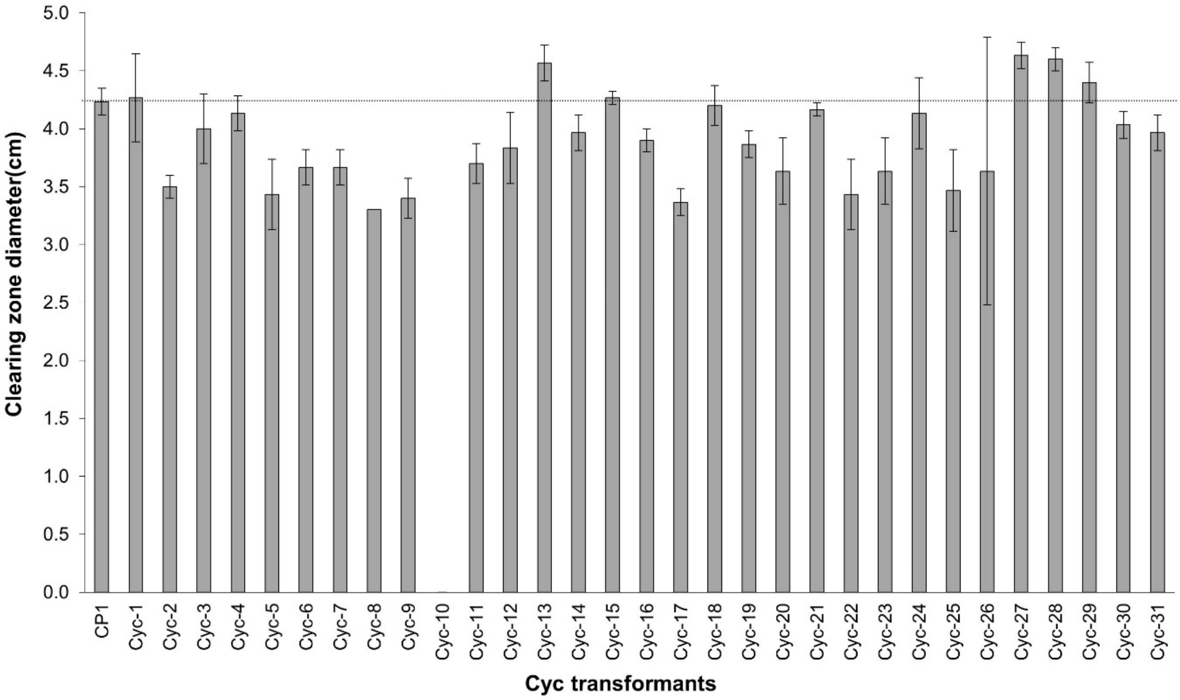

Supplementary Figure 21. Clearing zones recorded on plate-based bioassays for *C. passeckerianus* strains transformed with plasmid pYES-hph-iCycgene.

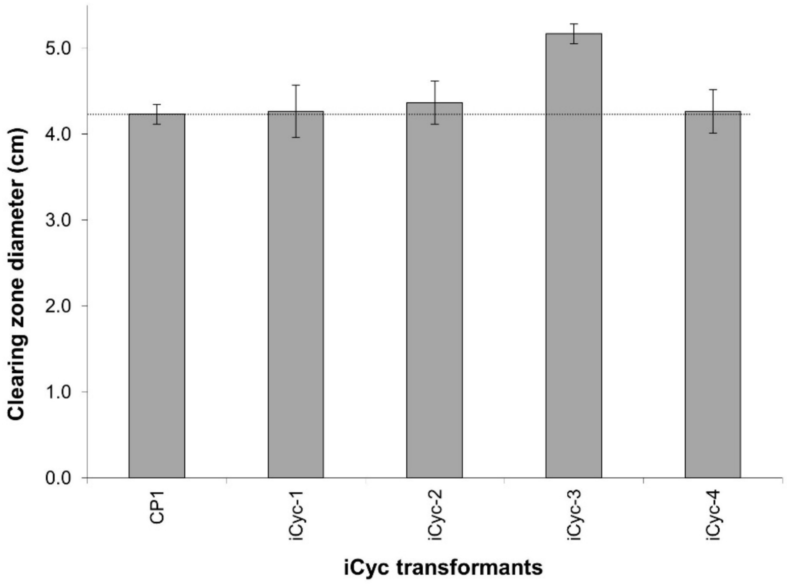

Supplementary Figure 22. Clearing zones recorded on plate-based bioassays for *C. passeckerianus* strains transformed with plasmid pYES-hph-p450-1.

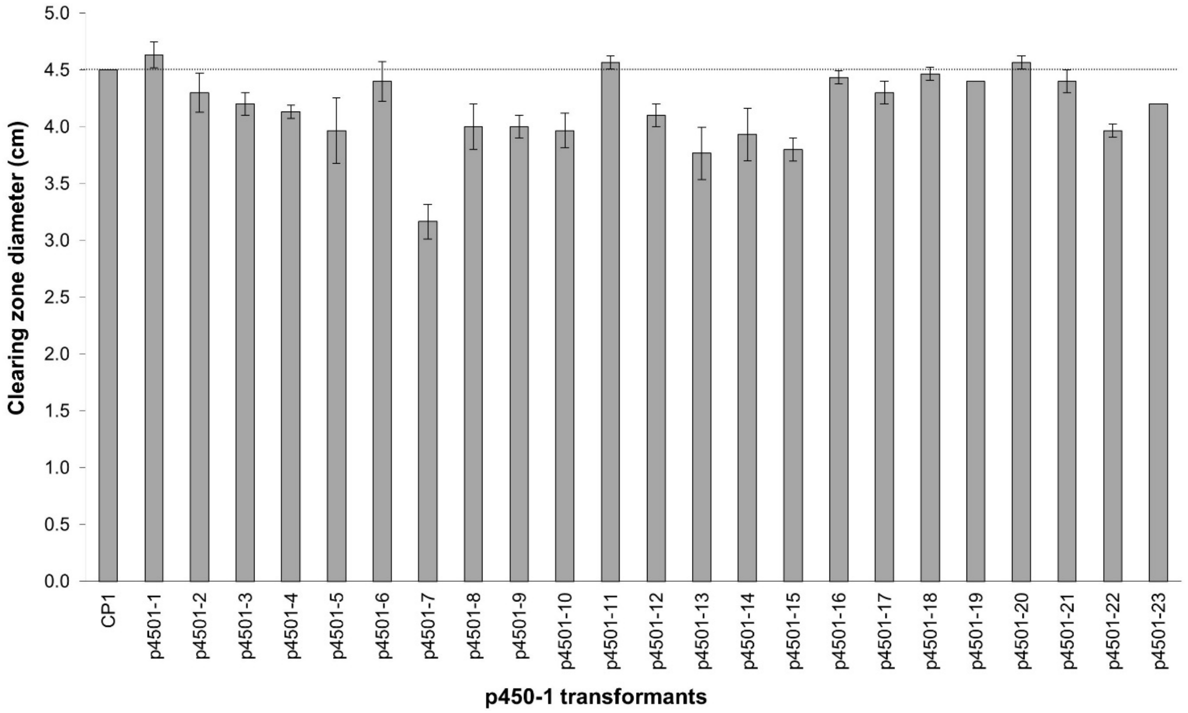

Supplementary Figure 23. Clearing zones recorded on plate-based bioassays for *C. passeckerianus* strains transformed with plasmid pYES-hph-ip450-1.

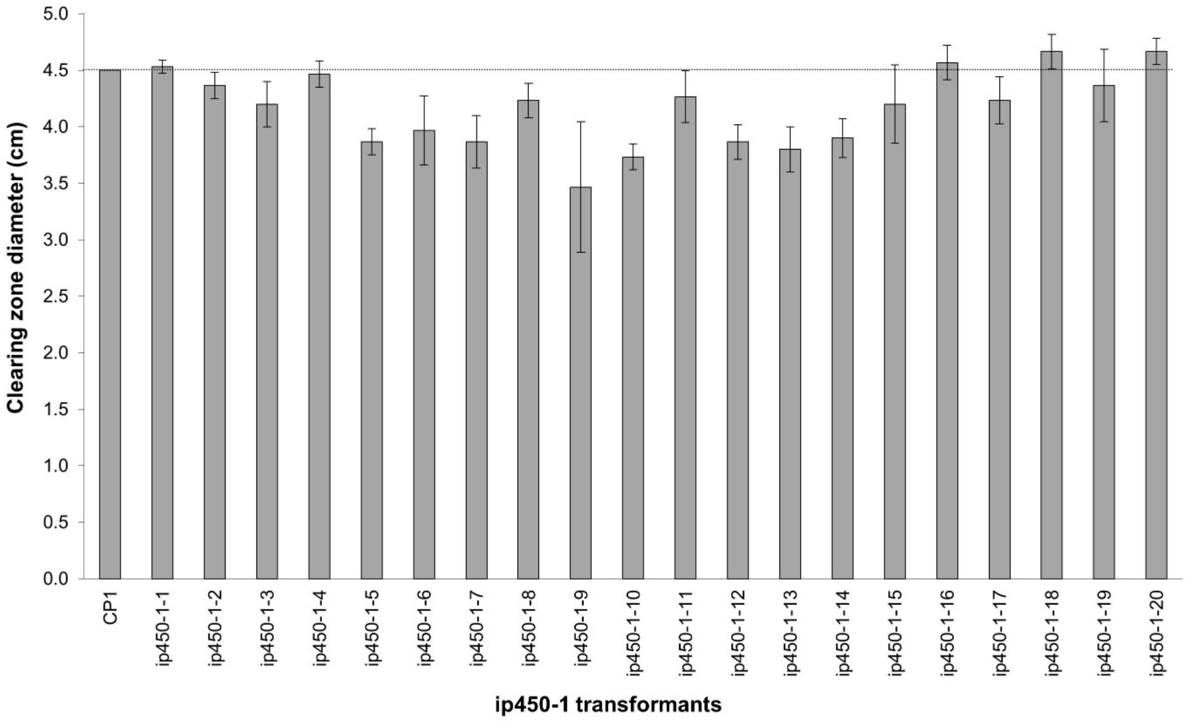

Supplementary Figure 24. Clearing zones recorded on plate-based bioassays for *C. passeckerianus* strains transformed with plasmid pYES-hph-p450-2.

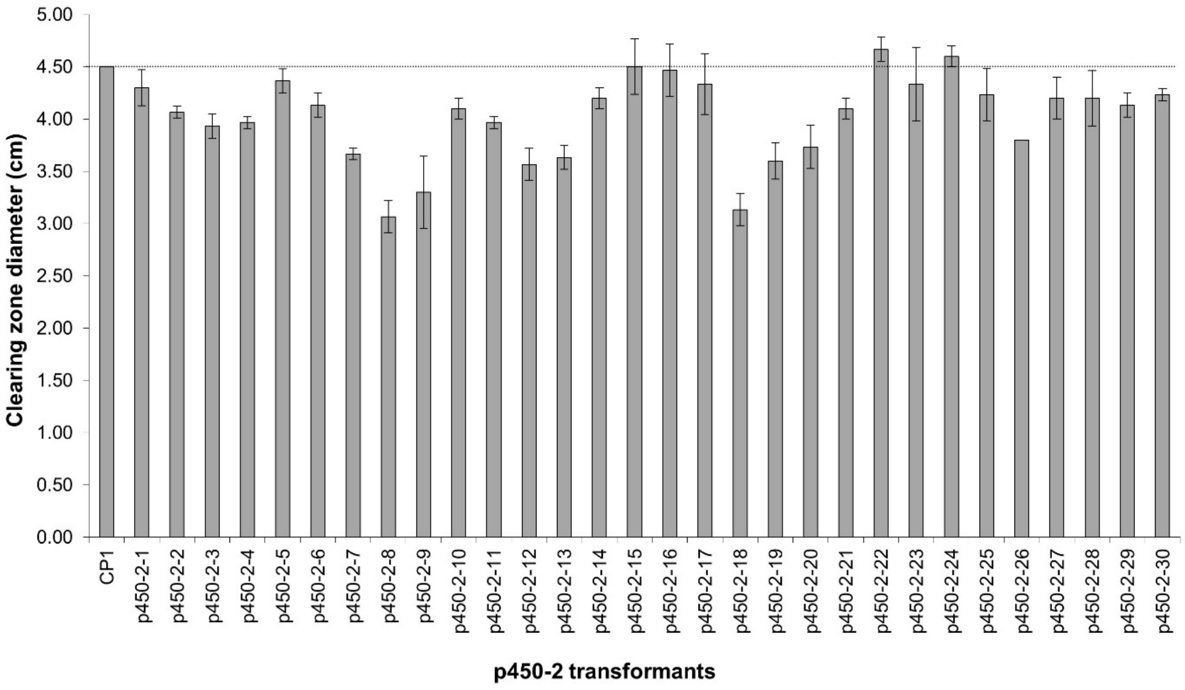

Supplementary Figure 25. Clearing zones recorded on plate-based bioassays for *C. passeckerianus* strains transformed with plasmid pYES-hph-ip450-2.

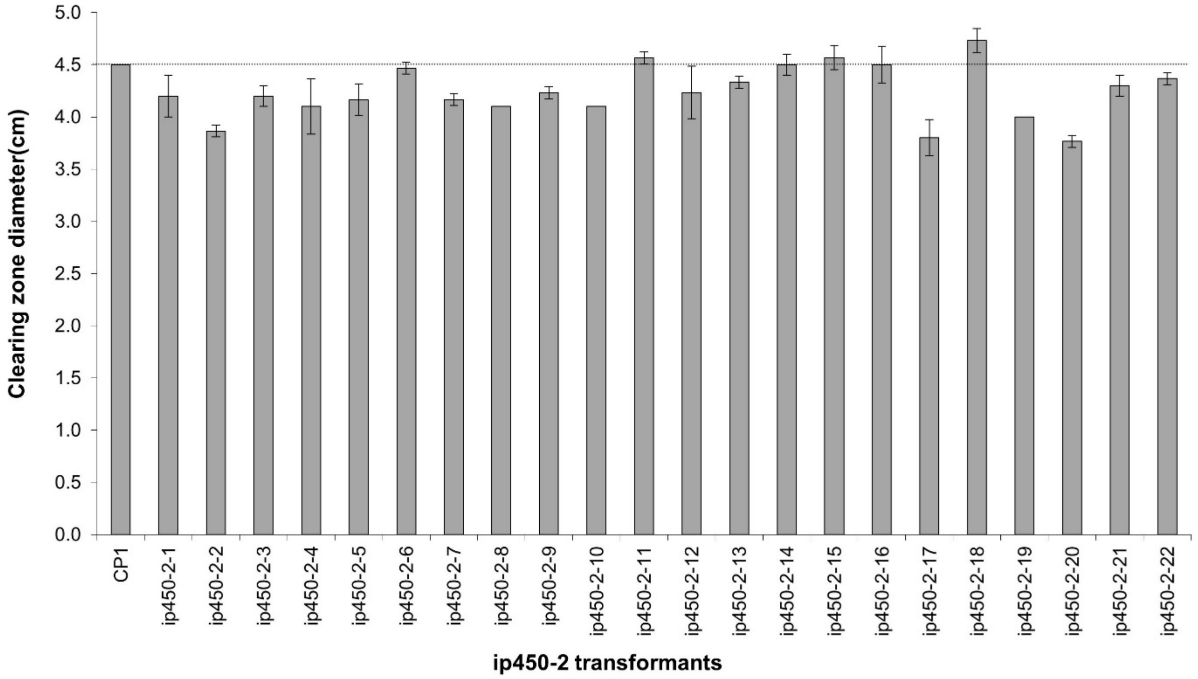

Supplementary Figure 26. Clearing zones recorded on plate-based bioassays for *C. passeckerianus* strains transformed with plasmid pYES-hph-ip450-3.

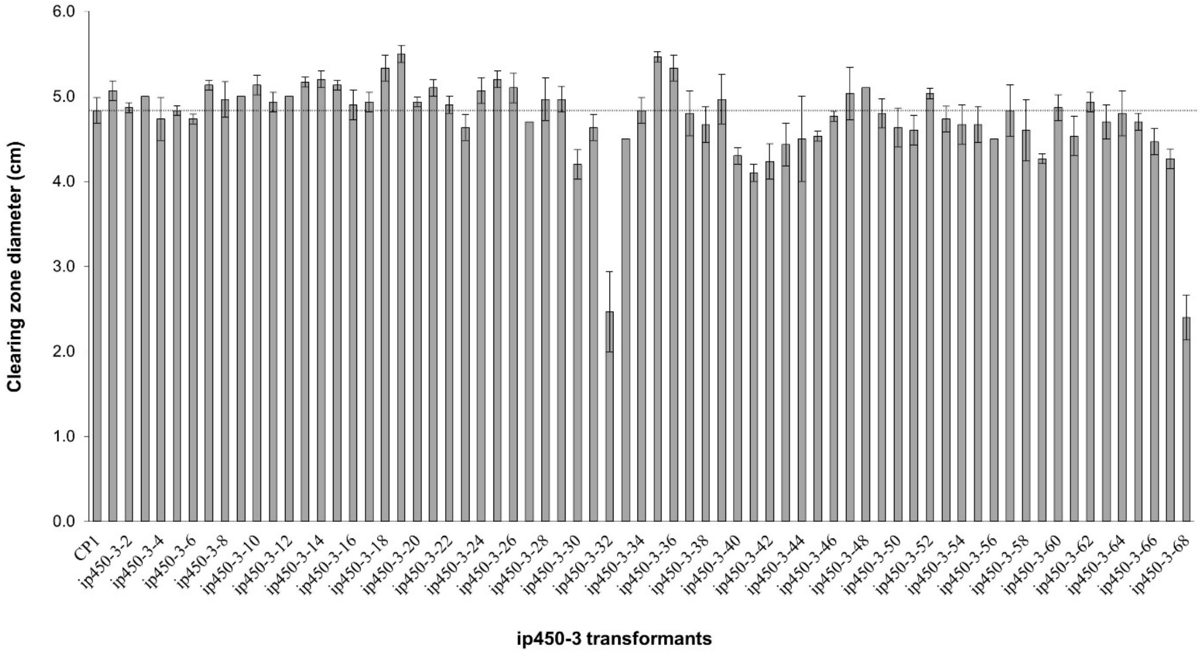

.

Supplementary Figure 27. Clearing zones recorded on plate-based bioassays for *C. passeckerianus* strains transformed with plasmid pYES2-hph-pleurocluster (Strains from 1 to 60).

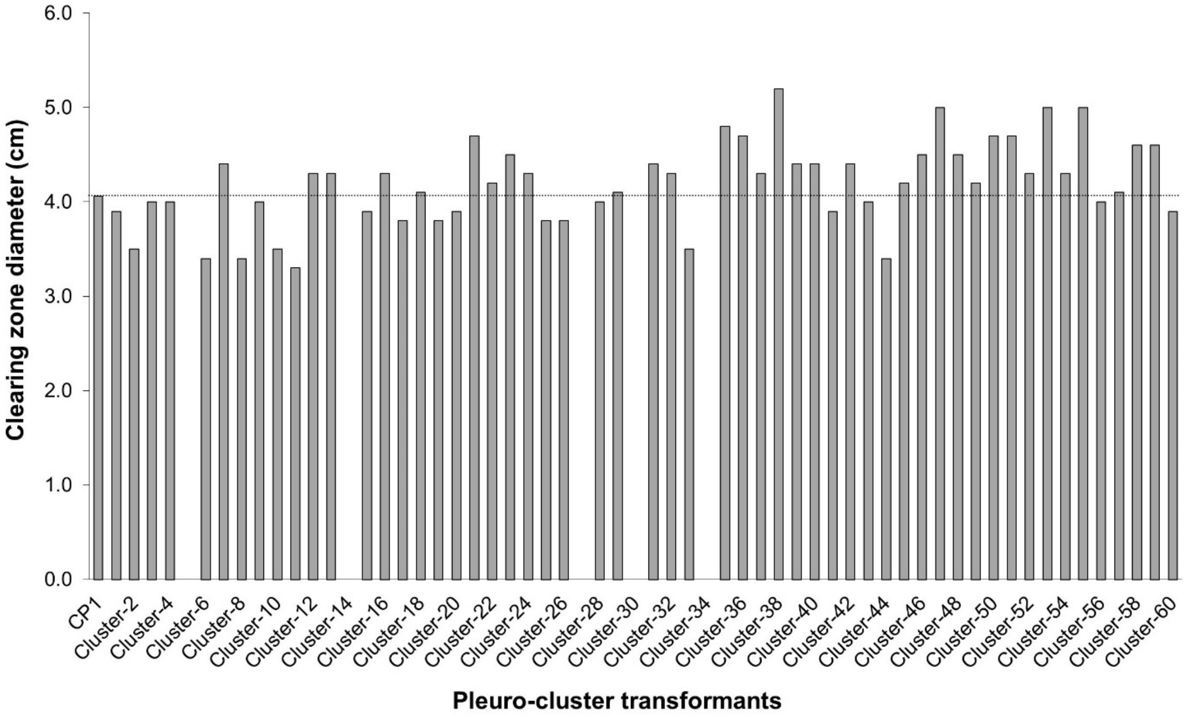

Supplementary Figure 28. Clearing zones recorded on plate-based bioassays for *C. passeckerianus* strains transformed with plasmid pYES2-hph-pleurocluster (Strains from 61 to 119).

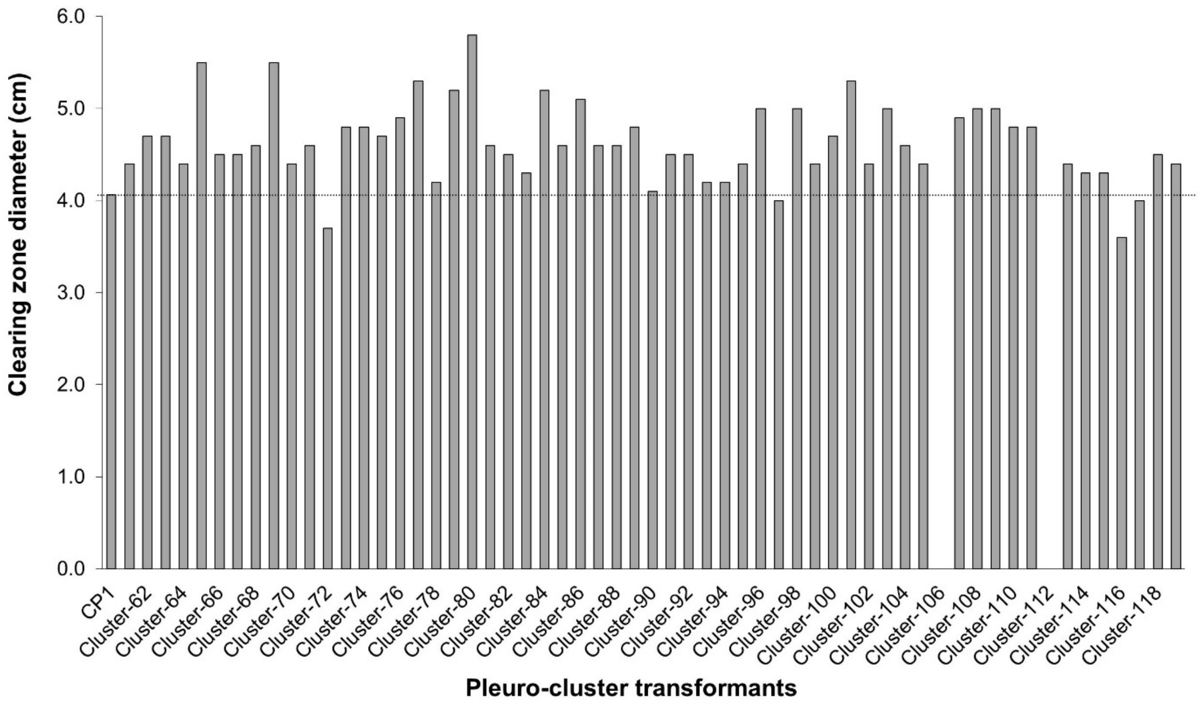

Supplementary Figure 29. Bioassays to evaluate antimicrobial activity of *A. oryzae* seven-gene transformants. *A. oryzae* NSAR1 and *C. passeckerianus* CP1 were included as respectively negative and positive controls. Error bars show the standard deviation of triplicate measurements for both colony diameter (bar in black) and inhibition zone diameter (bar in grey).

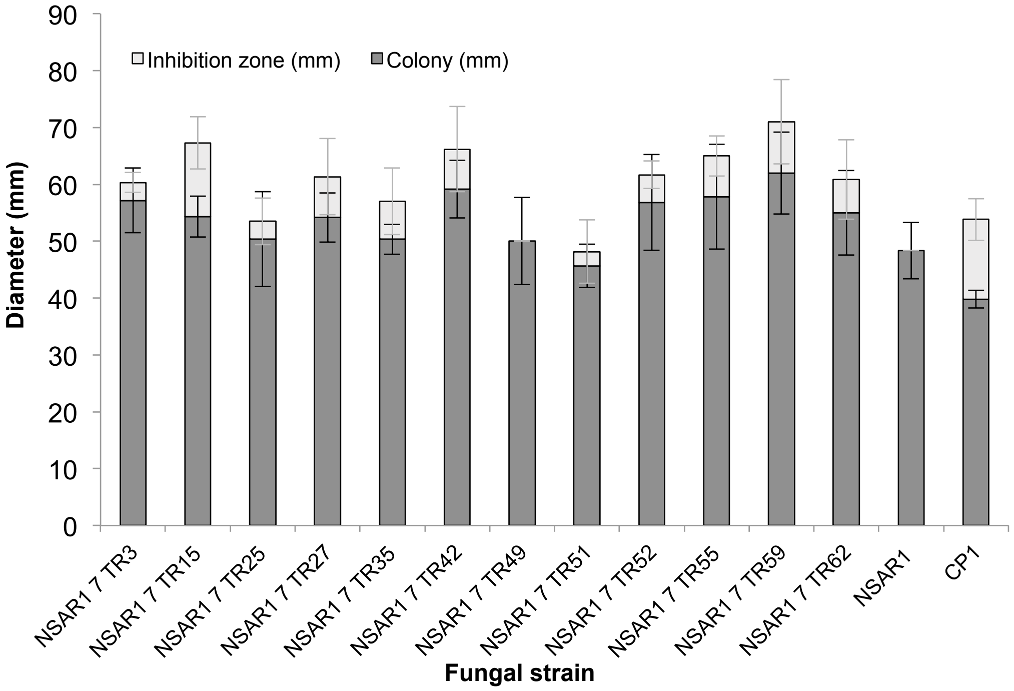

Supplementary Figure 30. RT-PCR analysis on *A. oryzae* seven-gene transformants for expression of the genes of the pleuromutilin cluster: *Pl-ggs* (i), *Pl-cyc* (ii), *Pl-p450-1* (iii), *Pl-p450-2* (iv), *Pl-p450-3* (v), *Pl-atf* (vi), *Pl-sdr* (vii). For each gel, lane 1: 5 µL of hyperladder I (Bioline); lanes 2-13: cDNA from *A. oryzae* seven-gene transformants; lane 14: cDNA from *A. oryzae* NSAR1; lane 15: plasmid used for transformation of *A. oryzae* containing the respective gene tested in RT-PCR (pTYGSargGGSCyc, pTYGSadeP450s, or pTYGSbarATFSDR); lane 16: water as template.

i. Gene amplified: *Pl-ggs*. Primers used: GGS FF/GGS RR. Expected amplicon size: 1,053 bp.

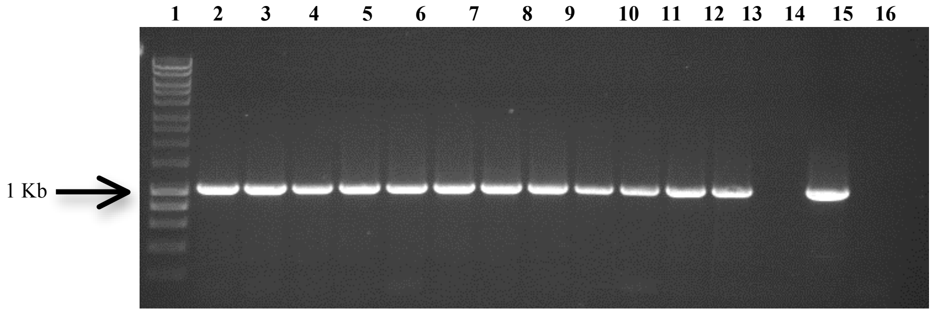

ii. Gene amplified: *Pl-cyc*. Primers used: Cyclase FF/Cyclase RR. Expected amplicon size:

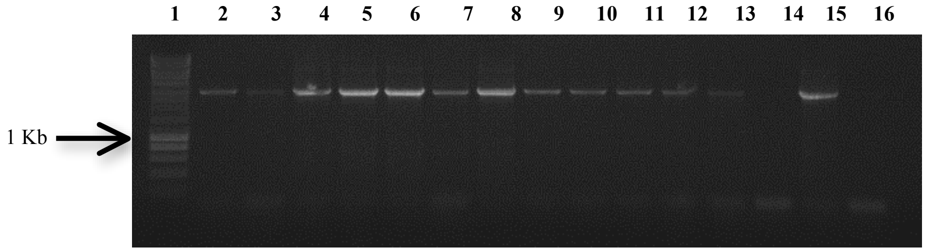

2,880 bp.

iii. Gene amplified: *Pl-p450-1*. Primers used: P450-1 FF/P450-1RR. Expected amplicon size: 1,572 bp.

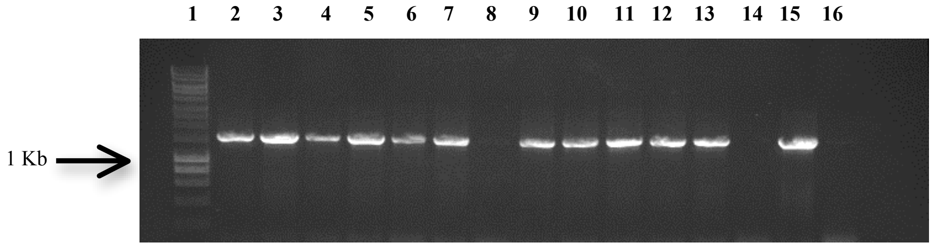

iv. Gene amplified: *Pl-p450-2*. Primers used: P450-2 FF/P450-2RR. Expected amplicon size:

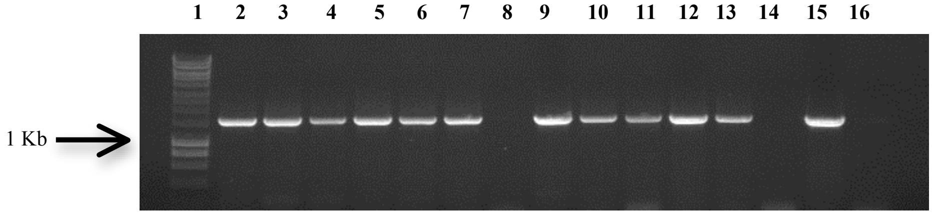

1,578 bp.

v. Gene amplified: *Pl-p450-3*. Primers used: P450-3 FF/P450-3RR. Expected amplicon size:

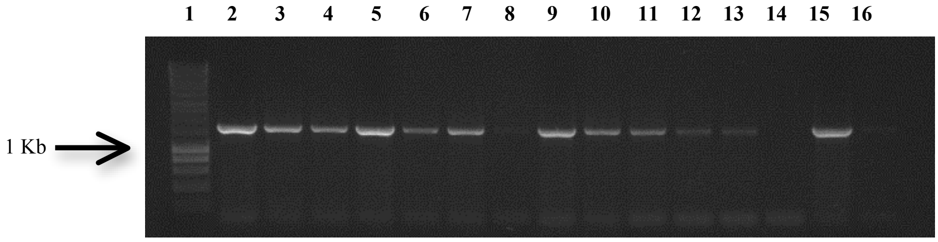

1,589 bp.

vi. Gene amplified: *Pl-atf*. Primers used: ATF FF/ATF RR. Expected amplicon size: 1,134 bp.

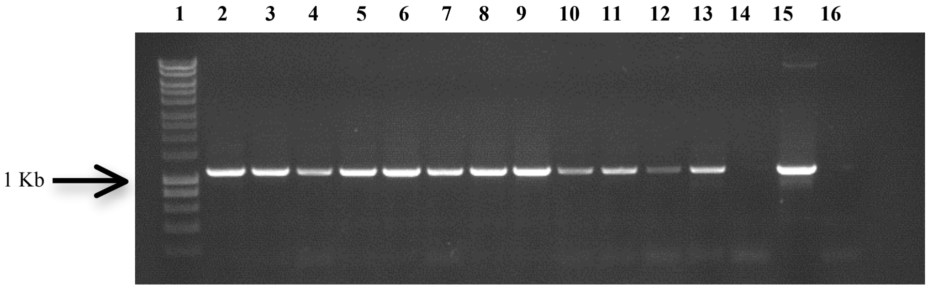

vii. Gene amplified: *Pl-sdr*. Primers used: SDR FF/SDR RR. Expected amplicon size: 782 bp.

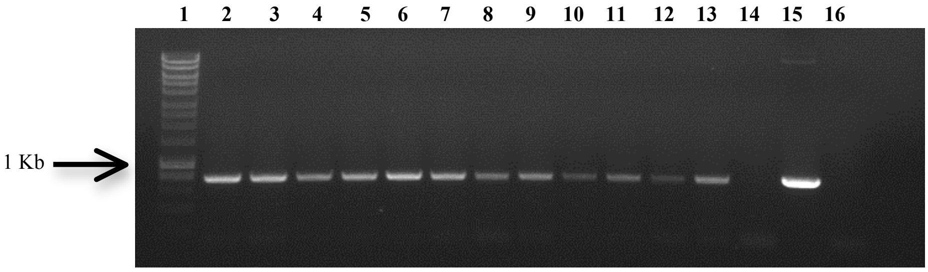

Supplementary Figure 31. ^{1}H-NMR spectrum of **1** in $CDCl_3$ (500 MHz). The signal observed at 7.26 ppm is from hydrogens contained in $CDCl_3$.

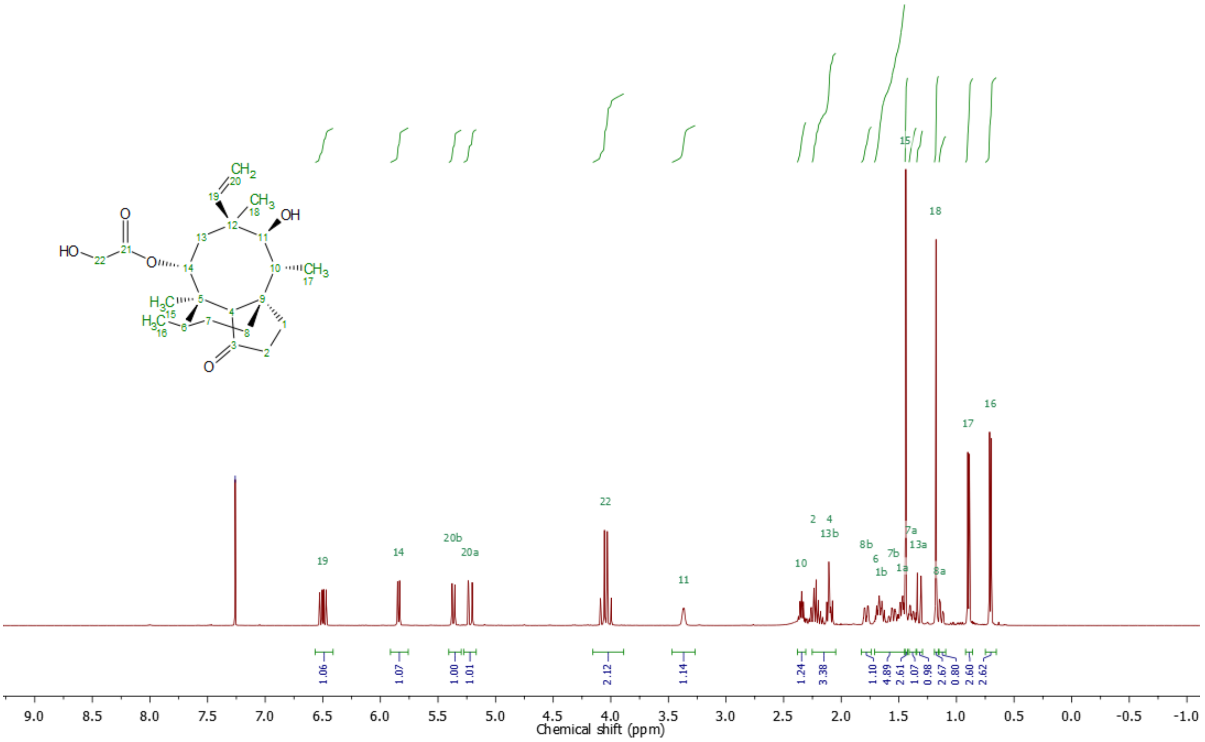

Supplementary Figure 32. ^{13}C-NMR spectrum of **1** in $CDCl_3$ (125 MHz). The signal observed at 77.16 ppm is from the carbon contained in $CDCl_3$.

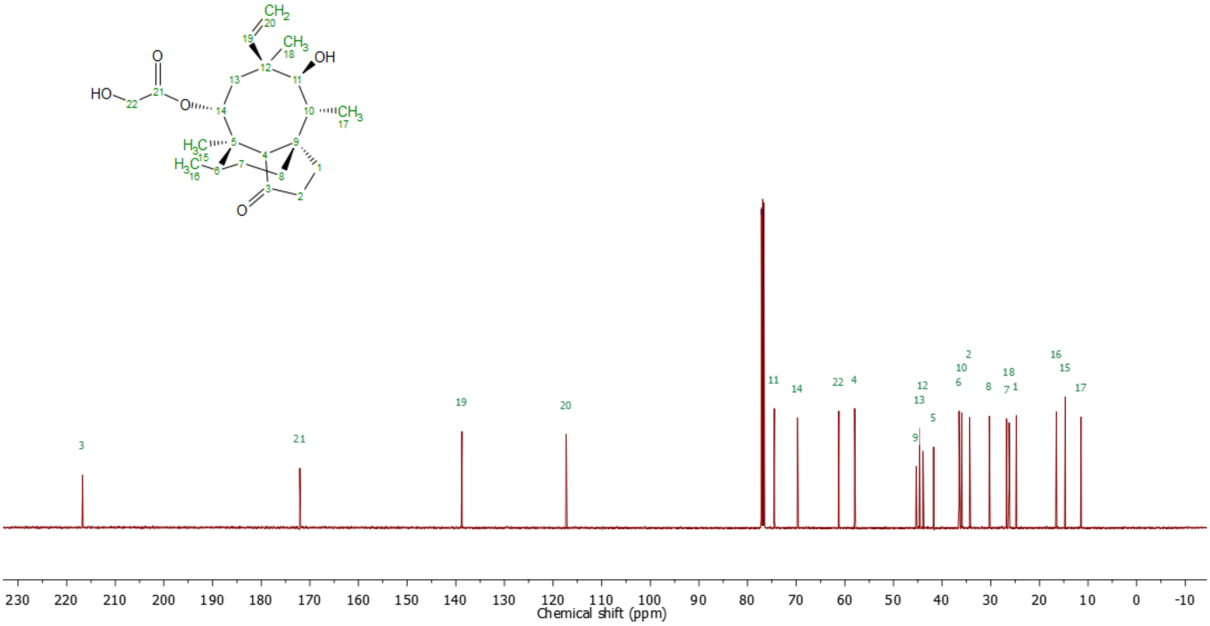

Supplementary Figure 33. ^{1}H-NMR spectrum of authentic pleuromutilin in $CDCl_3$ (500 MHz). The signal observed at 7.26 ppm is from hydrogens contained in $CDCl_3$.

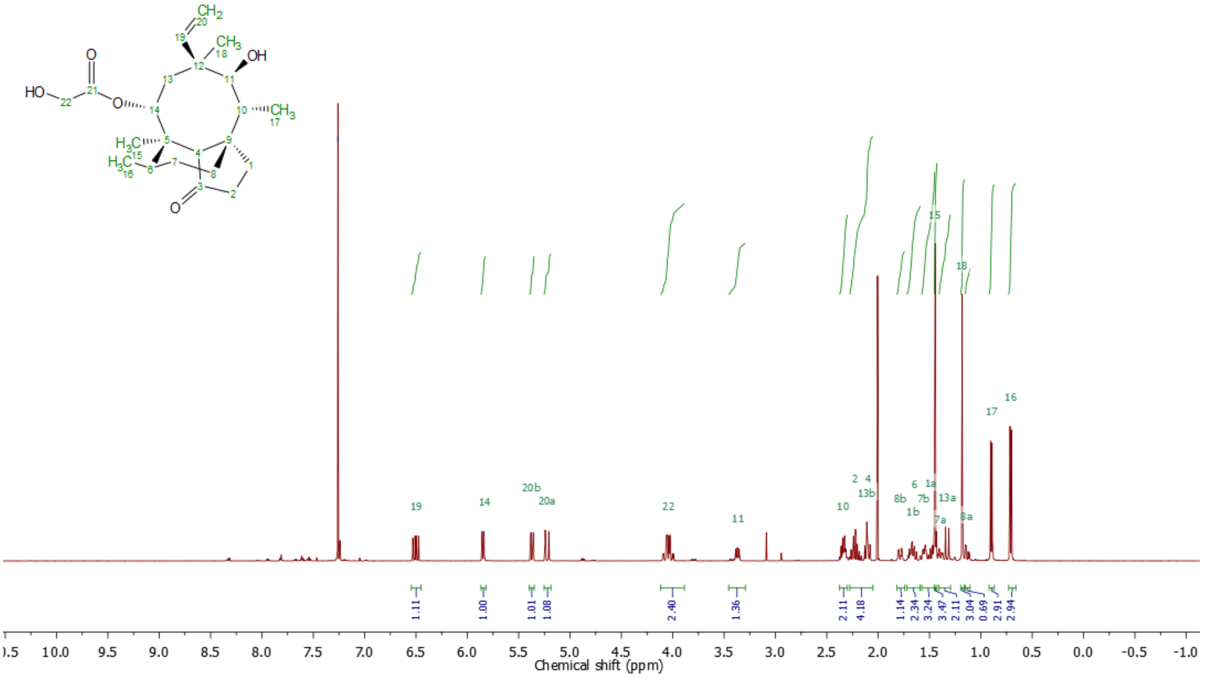

Supplementary Figure 34. ^{13}C-NMR spectrum of authentic pleuromutilin in $CDCl_3$ (125 MHz). The signal observed at 77.16 ppm is from the carbon contained in $CDCl_3$.

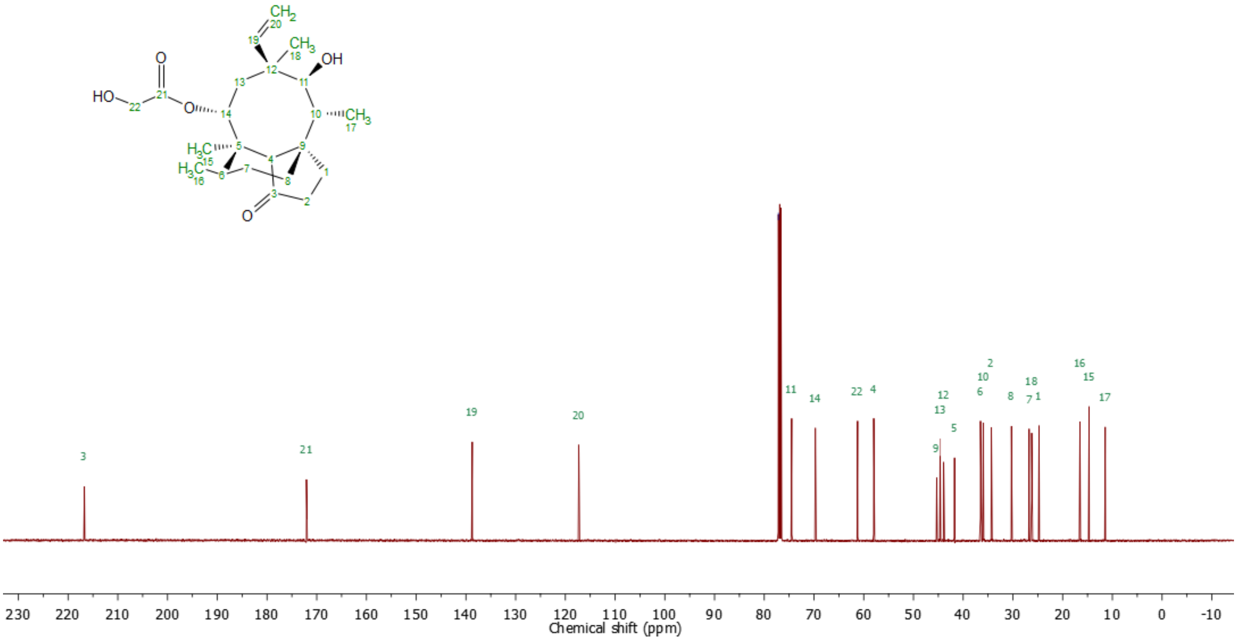

Supplementary Table 2. NMR data of **1** in $CDCl_3$. Experimental data for **1** (isolated from *A. oryzae* transformants) are reported in the left part of the table, whereas the reference data [3] are reported in the right part of the table. The molecular formula of **1** was established by high-resolution mass spectrometry (ESIHRMS: *m/z* 401.2291 $[M+Na]^+$, calculated for $C_{22}H_{34}O_5Na^+$: 401.2298, Δ = 0.7 mmu).

	Experimental data for 1				Reference data for pleuromutilin			
**#**	**$^{13}C(\delta)$**	**$^{1}H(\delta)$**	***J* Coupling (Hz)**	**Protons**	**$^{13}C(\delta)$**	**$^{1}H(\delta)$**	***J* Coupling (Hz)**	**Protons**
**1**	24.8	1.47	m	1	24.8	1.37-1.74	m	2
		1.67	m	1				
**2**	34.4	2.16-2.29	m	2	34.4	2.19-2.30	m	2
**3**	216.8				216.9			
**4**	58.1	2.07-2.15	m	1	58.1	2.07-2.18	m	1
**5**	41.8				41.8	-	-	-
**6**	36.6	1.68	m	1	36.6	1.37-1.74	m	1
**7**	26.8	1.37-1.43	m	1	26.8	1.37-1.74	m	2
		1.55	m	1				
**8**	30.4	1.11-1.17	m	1	30.4	1.14	dd, 13.8, 4.5	1
		1.80	dq, 14.5, 3.1	1		1.80	dd, 14.3, 3.0	1
**9**	45.4				45.4			
**10**	36.0	2.36	p, 7.0	1	36.0	2.31-2.41	m	1
**11**	74.6	3.38	d, 6.4	1	74.6	3.38	d, 6.5	1
**12**	44.0				44.0			
**13**	44.7	1.34	d, 16.1	1	44.7	1.34	d, 16.3	1
		2.07-2.15	m	1		2.07-2.18	m	1
**14**	69.8	5.85	d, 8.5	1	69.8	5.86	d, 8.5	1
**15**	14.8	1.45	s	3	14.8	1.45	s	3
**16**	16.6	0.72	d, 7.1	3	16.6	0.72	d, 6.8	3
**17**	11.5	0.91	d, 7.0	3	11.5	0.91	d, 7.0	3
**18**	26.3	1.19	s	3	26.3	1.19	s	3
**19**	138.8	6.51	dd, 17.4, 11.0	1	138.8	6.51	dd, 17.3, 11.0	1
**20**	117.4	5.23	dd, 17.4, 1.5	1	117.4	5.23	dd, 17.3, 1.3	1

		5.38	dd, 11.0, 1.5	1		5.38	dd, 11.0, 1.3	1
**21**	172.2				172.2			
**22**	61.3	4.05	dd, 29.3, 16.3	2	61.3	3.89-4.20	m	2

NMR spectroscopy was used to confirm the identity of **2**. ^{1}H-NMR (**Supplementary Fig. 35**) and ^{13}C-NMR (**Supplementary Fig. 36**) spectra were obtained from purified **2** from engineered *A. oryzae*. No reference was found in literature for 14-O-acetyl-mutilin, therefore a full-range of NMR analyses was carried out on **2**, including HSQC (**Supplementary Fig. 37**), HMBC (**Supplementary Fig. 38**) and 2D-COSY (**Supplementary Fig. 39**).

Supplementary Figure 35. ^{1}H-NMR spectrum of **2** in $CDCl_3$ (500 MHz). The signal observed at 7.26 ppm is from hydrogens contained in $CDCl_3$.

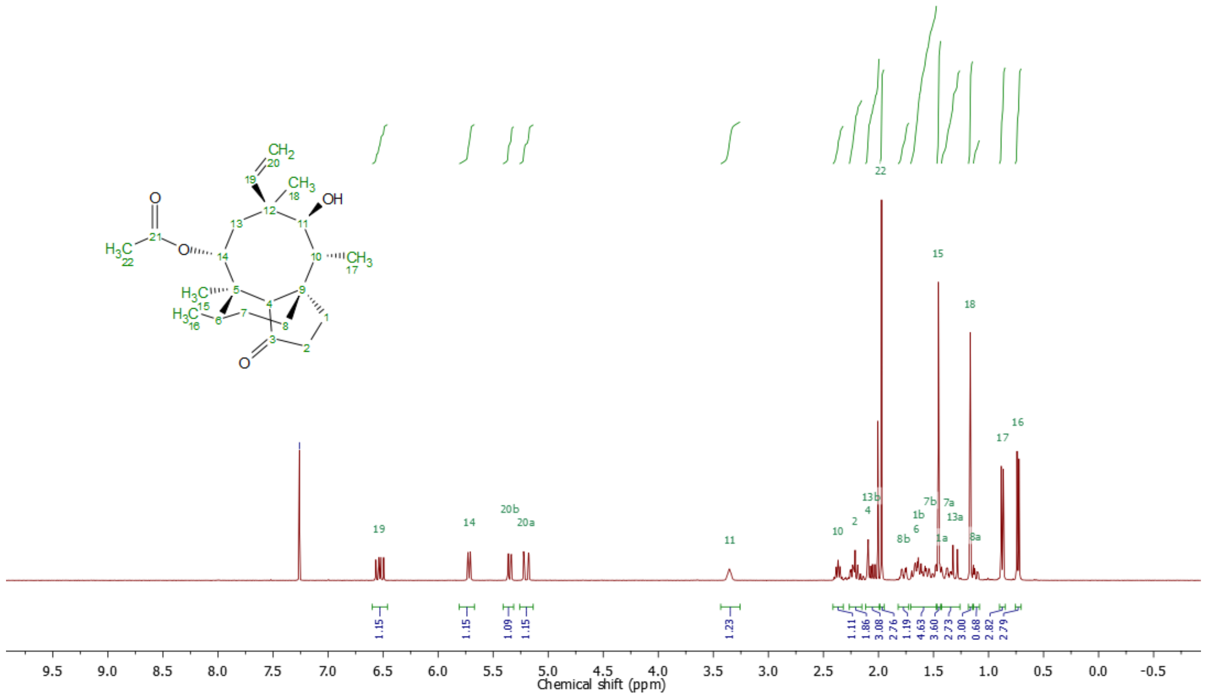

Supplementary Figure 36. ^{13}C-NMR spectrum of **2** in $CDCl_3$ (125 MHz). The signal observed at 77.16 ppm is from the carbon contained in $CDCl_3$.

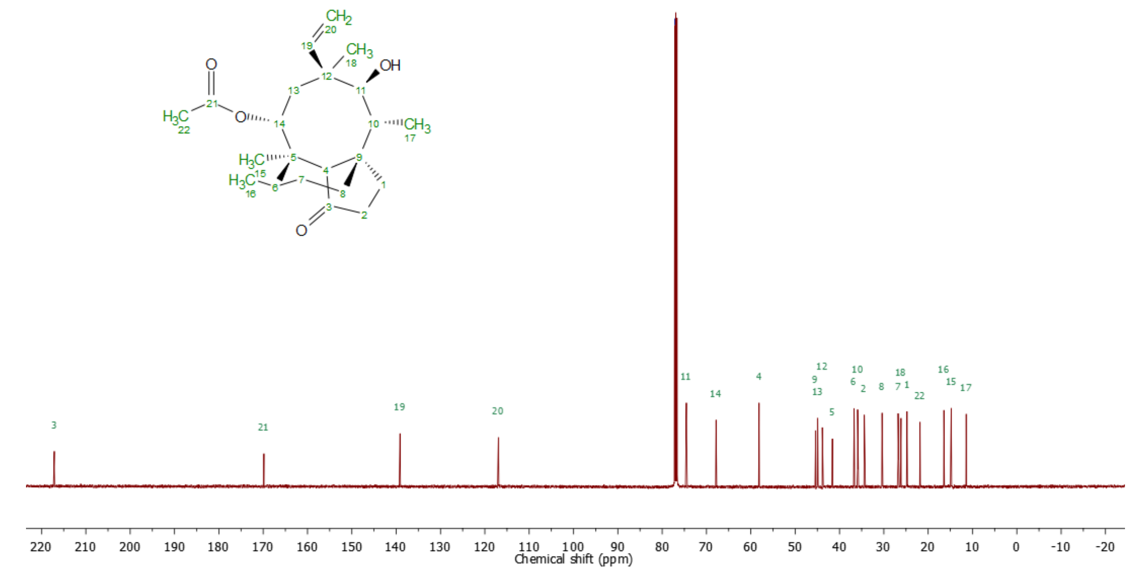

Supplementary Figure 37. HSQC spectrum of **2** in $CDCl_3$ (500 MHz).

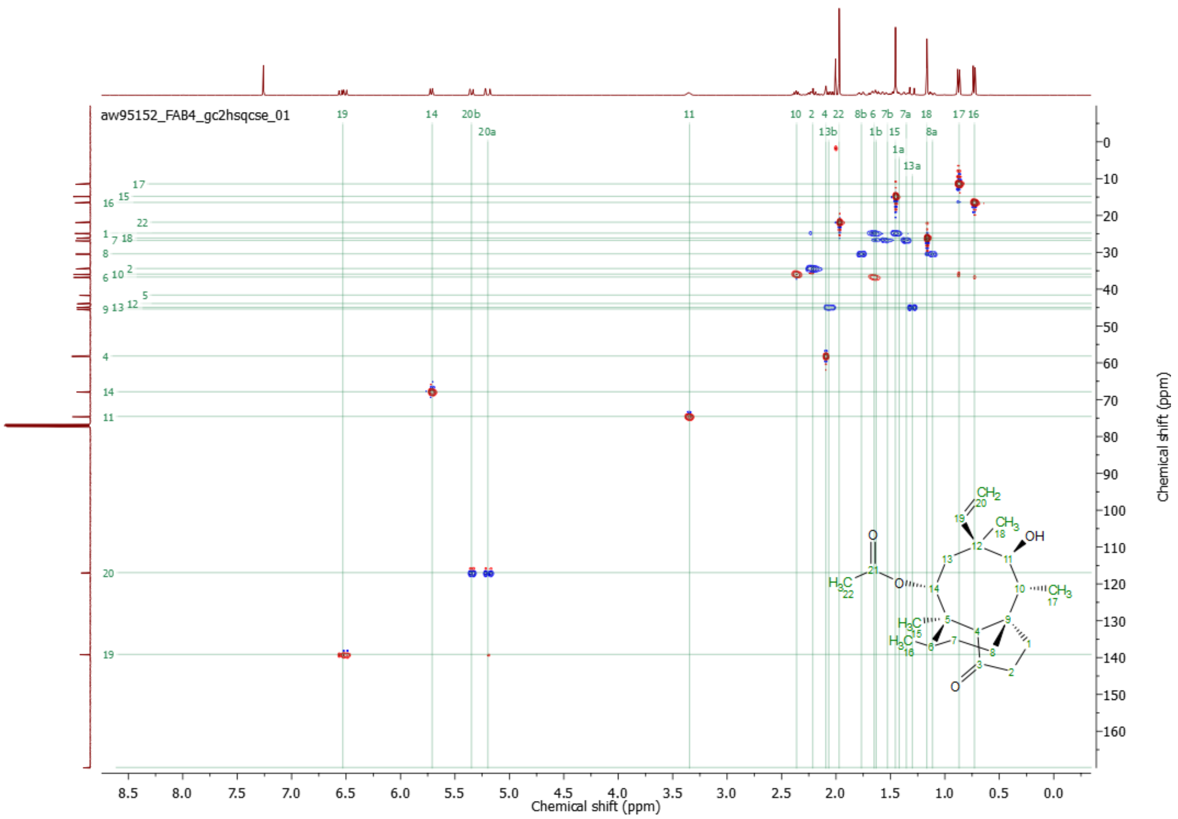

Supplementary Figure 38. HMBC spectrum of **2** in $CDCl_3$ (500 MHz).

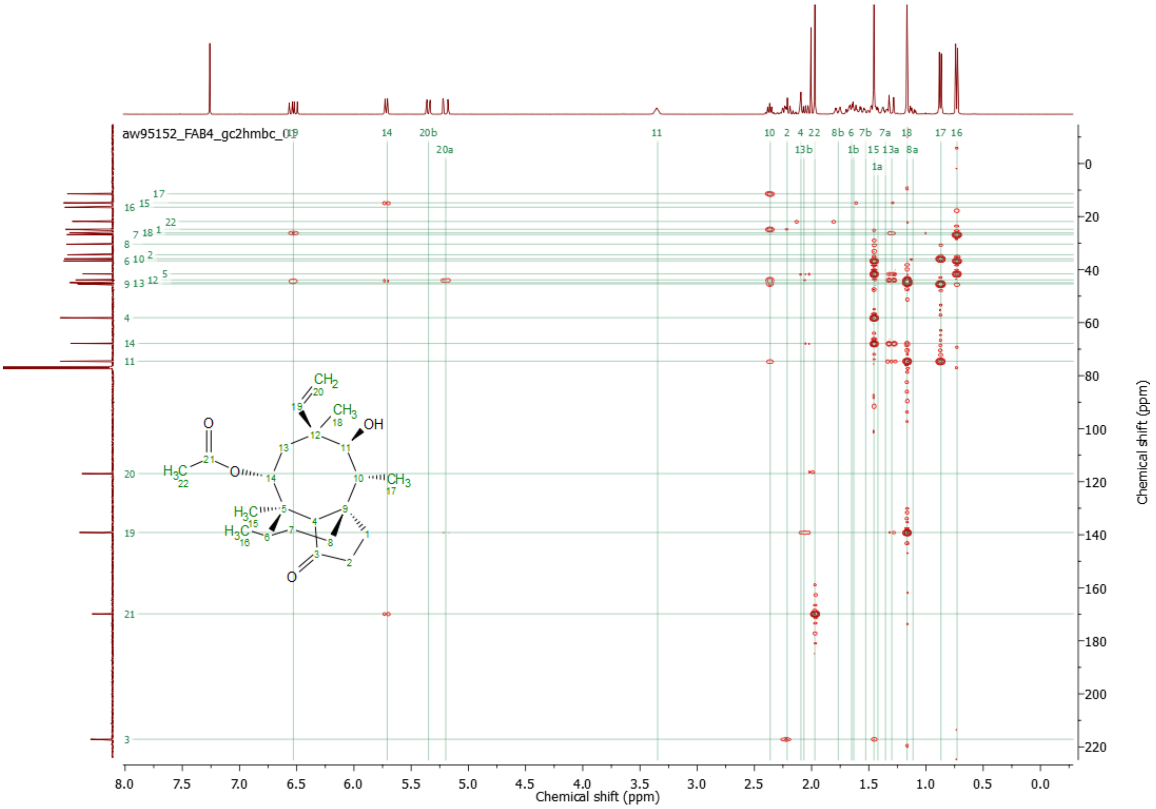

Supplementary Figure 39. 2D-COSY spectrum of **2** in $CDCl_3$ (500 MHz).

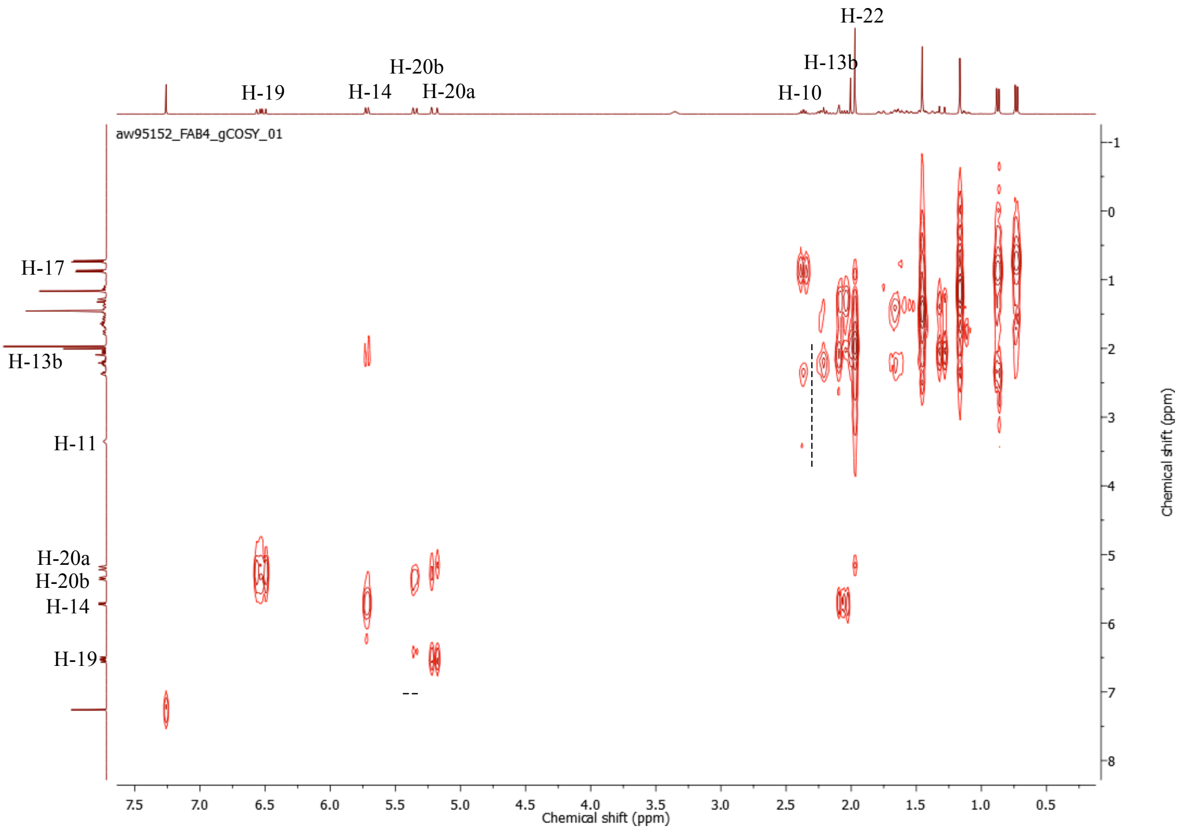

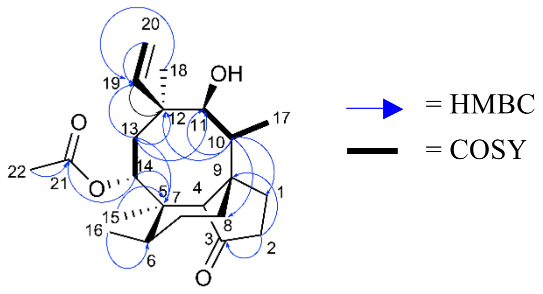

14-O-acetyl-mutilin (**2**)

Supplementary Table 3. NMR data of **2** in $CDCl_3$. The molecular formula of **2** was established by high-resolution mass spectrometry (ESIHRMS: *m/z* 385.2346 $[M+Na]^+$, calculated for $C_{22}H_{34}O_4Na^+$: 385.2349, Δ = 0.3 mmu).

#	$^{13}C(\delta)$	HSQC	$^{1}H(\delta)$	*J* Coupling (Hz)	Protons	HMBC	COSY
**1**	24.9	CH_2	1.45	m	1		
			1.64	m	1	C-2, C-8	
**2**	34.5	CH_2	2.23	m	2	C-1, C-3, C-4, C-9	
**3**	217.2						
**4**	58.2	CH	2.07	m	1	C-3, C-5, C-8, C-14	
**5**	41.7						
**6**	36.8	CH	1.64	m	1	C-7, C-8	
**7**	26.9	CH_2	1.34	m	1		
			1.53	m	1		
**8**	30.5	CH_2	1.12	m	1		
			1.77	m	1	C-22	
**9**	45.5						
**10**	36.0	CH	2.37	p, 7.0	1		H-17
**11**	74.6	CH	3.36	m	1	C-1, C-8, C-11, C-12, C-13, C-17	H-10
**12**	44.0						
**13**	45.0	CH_2	1.30	d, 16.0	1	C-5, C-11, C-12, C-14, C-18, C-19	
			2.07	m	1	C-5, C-18, C-19	H-14
**14**	67.9	CH	5.72	d, 8.5	1	C-5, C-6, C-13, C-15	
**15**	14.9	CH_3	1.45	s	3	C-3, C-4, C-5, C-6, C-14,	
**16**	16.6	CH_3	0.73	d, 7.3	3	C-5, C-6, C-7	
**17**	11.5	CH_3	0.87	d, 7.0	3	C-9, C-10, C-11	H-10
**18**	26.2	CH_3	1.17	s	3	C-11, C-13, C-19	
**19**	139.2	CH	6.53	dd, 17.4, 11.0	1	C-11, C-12, C-18	H20a, H-20b
**20**	117.0	CH_2	5.20	dd, 17.4, 1.6	1	C-12, C-19	H-19
			5.35	dd, 11.0, 1.6	1	C-12	H-19

**21**	169.6						
**22**	21.9	CH_3	1.97	s		3	C-21

Supplementary Table 4. Summary of transgene copy number through Southern Blot analysis.

*= Interpretation of copy number not confident due to non-specific binding.

	Gene probed (genes present on the same plasmid)			
**Strain**	***Cyclase*** (*ggs*)	***P450-2***(*p450-1, p450-3*)	***SDR***	***ATF***
A. oryzae **NSAR1 7 TR27**	1	1	1	1
A. oryzae **NSAR1 7 TR51**	1	2	4	3
A. oryzae **NSAR1 7 TR52**	1	3	1	-*

1. Cummings, W.J., Celerin, M., Crodian, J., Brunick, L.K. & Zolan, M.E. Insertional mutagenesis in *Coprinus cinereus*: use of a dominant selectable marker to generate tagged, sporulation-defective mutants. *Curr Genet* **36**, 371-382 (1999).
2. Kilaru, S., Collins, C.M., Hartley, A.J., Bailey, A.M. & Foster, G.D. Establishing molecular tools for genetic manipulation of the pleuromutilin-producing fungus *Clitopilus passeckerianus*. *Applied and Environmental Microbiology* **75**, 7196-7204 (2009).
3. Fazakerley, N.J., Helm, M.D. & Procter, D.J. Total Synthesis of (+)-Pleuromutilin. *Chemistry – A European Journal* **19**, 6718-6723 (2013).
